# The optimized core peptide derived from CABIN1 efficiently inhibits calcineurin-mediated T-cell activation

**DOI:** 10.1038/s12276-022-00772-6

**Published:** 2022-05-12

**Authors:** Sangho Lee, Han-Teo Lee, Young Ah Kim, Il-Hwan Lee, Seong-Jun Kang, Kyeongpyo Sim, Chung-Gyu Park, Kyungho Choi, Hong-Duk Youn

**Affiliations:** 1 National Creative Research Center for Epigenome Reprogramming Network, Department of Biomedical Sciences, Ischemic/Hypoxic Disease Institute, Seoul National University College of Medicine, Seoul, 03080 Republic of Korea; 2 Department of Molecular Medicine & Biopharmaceutical Sciences, Graduate School of Convergence Science, Seoul National University, Seoul, 03080 Republic of Korea; 3 Department of Biomedical Science, Seoul National University College of Medicine, Seoul, 03080 Republic of Korea; 4 Department of Microbiology and Immunology, Seoul National University College of Medicine, Seoul, 03080 Republic of Korea

**Keywords:** Protein-protein interaction networks, Cell death and immune response

## Abstract

The *C*-terminal fragment of CABIN1 interacts with calcineurin and represses the transcriptional activity of the nuclear factor of activated T cells (NFAT). However, the specific sequences and mechanisms through which it binds to calcineurin are unclear. This study determined that decameric peptide (CABIN1 residues 2146–2155) is minimally required for binding to calcineurin. This peptide contains a unique “PPTP” *C*-terminal sequence and a “PxIxIT” *N*-terminal motif. Furthermore, p38MAPK phosphorylated the threonine residue of the “PPTP” sequence under physiological conditions, dramatically enhancing the peptide’s binding affinity to calcineurin. Therefore, the CABIN1 peptide inhibited the calcineurin-NFAT pathway and the activation of T cells more efficiently than the VIVIT peptide without affecting calcineurin’s phosphatase activity. The CABIN1 peptide could thus be a more potent calcineurin inhibitor and provide therapeutic opportunities for various diseases caused by the calcineurin-NFAT pathway.

## Drug development: Potential pathway inhibitor for cancer and autoimmune diseases

A peptide with therapeutic potential binds strongly to the cellular enzyme calcineurin and may prove valuable in anti-cancer and autoimmune disease treatments. Many cancers and autoimmune diseases are linked with overactivation of a key calcineurin-related pathway which is heavily involved in T cell activation. This pathway has long been a therapeutic target, but existing drugs show problems with stability and delivery, and can cause serious side effects. One known inhibitor of calcineurin is the protein CABIN1, but precisely how well it binds and how useful it may be is unclear. Now, Hong-Duk Youn at Seoul National University College of Medicine, South Korea, and co-workers have identified how one specific peptide from CABIN1 binds strongly to calcineurin. The CABIN1 peptide was stable and displayed greater efficiency at inhibiting calcineurin than another recently identified peptide candidate.

## Introduction

Calcineurin is calcium and calmodulin-dependent serine/threonine phosphatase that plays a pivotal role in diverse organs or cells, such as the brain, cardiovascular and skeletal muscle, pancreatic β cells, and T lymphocytes^1–4^. Over the past few decades, many substrates and inhibitors of calcineurin containing conserved motifs for interaction with calcineurin, “PxIxIT” and/or “﻿LxVP,” have been identified. For instance, nuclear factor of activated T cells (NFAT) family members are known calcineurin substrates containing both binding motifs^5^. In calcineurin-regulated NFATs (all NFAT family members except NFAT5), calcineurin dephosphorylates at least 13 conserved phosphorylation sites^6^. These sites are in the NFAT-homology region between the “PxIxIT” and “LxVP” motifs^7^. Several kinases, such as GSK3, CK1, p38, and JNK, phosphorylate these sites. These molecules cause conformational changes, expose nuclear export signals, and mask nuclear localization signals^8^.

Productive activation of T lymphocytes requires T-cell receptor (TCR) signaling and costimulatory signaling to act together. TCR signaling or costimulatory signaling alone leads to T-cell anergy or tolerance^9,10^. TCR stimulation activates the calcineurin-NFAT pathway by elevating intracellular Ca^2+^ concentrations^11^, and simultaneous stimulation of TCR/CD28 activates the PKC-MAPK and PKC-IKK-NFκB pathways^12,13^. The calcineurin-NFAT and PKC-MAPK pathways activate NFAT and AP-1 (Fos/Jun), which cooperate in the transcriptional activation of proinflammatory cytokines, such as interleukin (IL)-2, IL-3, IL-4, GM-CSF, and IFN-γ, thereby activating T lymphocytes^14^. However, PKC activation antagonizes the Ca^2+^-induced calcineurin-NFAT pathway, despite being important for T lymphocyte activation^15^. PKC-activated p38 phosphorylates nuclear NFATC2 (also known as NFAT1 or NFATp), thereby inducing the export of NFATC2 to the cytosol^16^.

The conventional calcineurin inhibitors FK506 and cyclosporin A have serious side effects. However, they are still widely used in autoimmune diseases and organ transplant patients due to their convenient dosing and effectiveness^17^. Although several alternative calcineurin inhibitors have been developed over the past few decades, none have become clinical treatments thus far. The VIVIT peptide, an optimized peptide derived from the conserved “PxIxIT” sequence of the NFAT family, blocks the interaction between calcineurin and NFAT without affecting calcineurin phosphatase activity^18^. Although this peptide showed potential as an alternative calcineurin inhibitor for various calcineurin-NFAT pathway-dependent diseases, it has a high IC_50_ value and drug delivery limitations^19^.

CABIN1 was first identified as an endogenous calcineurin inhibitor protein. Upon T-cell activation, the CABIN1 *C*-terminal fragment binds to and inhibits calcineurin, thereby blocking NFAT dephosphorylation and repressing *IL-2* promoter transcriptional activation^20^. Developing alternative calcineurin inhibitors using CABIN1 requires identifying the specific regions that interact with calcineurin. Although the *C*-terminus of CABIN1 contains a “PxIxIT” motif^19^, it is unknown whether this fragment’s calcineurin inhibitory effect is due to the “PxIxIT” motif. In this study, we inhibited the calcineurin-NFAT pathway using a short peptide from CABIN1. We identified the 10 amino acids required for interaction with calcineurin in the CABIN1 *C*-terminal fragment. This peptide inhibited the calcineurin-NFAT pathway more efficiently than did the VIVIT peptide, without affecting calcineurin phosphatase activity. We demonstrated that these results were due to the “PPTP” sequence following the “PxIxIT” motif. The binding affinity of the CABIN1 peptide to calcineurin depends on the phosphorylation of its ninth threonine residue (residue 2154 in endogenous CABIN1). Furthermore, p38MAPK is a putative upstream kinase of the CABIN1 peptide. Since the optimized CABIN1 peptide harbors an additional “PPTP” sequence, which is phosphorylated under physiological conditions, we suggest that the CABIN1 peptide could be a potent inhibitor of the calcineurin-NFAT pathway in vivo.

## Materials and methods

### Cell culture and transfection

We purchased HEK293T and Jurkat T (E6-1) cells from ATCC (Manassas, VA, USA). We cultured HEK293T cells in Dulbecco’s modified Eagle’s medium supplemented with 10% (v/v) fetal bovine serum (Gibco, Grand Island, NY, USA) and antibiotics at 37 °C and 5% CO_2_ and transfected them using polyethyleneimine (Polysciences, Warrington, FL, USA). We cultured Jurkat T cells in RPMI 1640 medium supplemented with 10% (v/v) fetal bovine serum (Gibco), 2 mM l-glutamine (Gibco), and antibiotics at 37 °C and 5% CO_2_. Following the manufacturer’s instructions, we transfected Jurkat T cells by electroporation using a Neon™ Transfection System (Thermo Fisher Scientific, Waltham, MA, USA).

### Plasmid DNA constructs

This study used the previously described calcineurin catalytic subunit (CNA) isoforms, CABIN1-14, CABIN1-15, tNFATC2, pG5-luc, and NFAT-luc^20^. We cloned the CNA isoforms into expression vectors such as pVP16, pRSET-A/B, and pCAG-Flag. We obtained oligonucleotides of CABIN1, VIVIT, and chimeric and substituted peptides from Macrogen (Seoul, Korea) or Cosmogenetech (Seoul, Korea), and annealed them for hybridization, and inserted them into expression vectors such as pM, pGEX4T-1, pCAG-HA-GST, and pCAG-HA-mCherry. We constructed the pCAG-EGFP-tNFATC2-IRES-mCherry-peptide by replacing the puromycin resistance gene following the IRES segment with the mCherry-tagged peptide. We confirmed all the constructs by sequencing.

### Reagents and antibodies

We purchased the anti-GAL4 DNA binding domain (GAL4 DBD) (RK5C1) antibody from Santa Cruz Biotechnology (Dallas, TX, USA) and anti-NFATC2, pan-CNA, and phospho-MAPK Substrates Motif [PXpTP] antibodies from Cell Signaling (Danvers, MA, USA). We purchased anti-β-actin (AC-15) and Flag (M2) antibodies and Anti-FLAG ® M2 Affinity Gel from Sigma–Aldrich (St. Louis, MO, USA). We obtained the anti-HA (16B12) antibody from BioLegend (San Diego, CA, USA) and Pierce™ Anti-HA Agarose from Thermo Fisher Scientific. We acquired phorbol myristate acetate (PMA) and ionomycin from Sigma–Aldrich, FK506 from InvivoGen (San Diego, CA, USA), and EO 1428 and TAK 715 from Tocris (Bristol, UK).

### Peptide synthesis

Peptron (Daejeon, Korea) synthesized the unlabeled peptides (CABIN1-L-1 [MAGFPPEITVTPPTP] and VIVIT 15-mer [MAGPHPVIVITGPHEE]). GenScript (Piscataway, NJ, USA) synthesized the biotin-labeled peptides (VEET [Biotin-AAAMAGPPHIVEETGPHVI], VIVIT 15-mer [Biotin-AAAMAGPHPVIVITGPHEE], CABIN1-L-1 [Biotin-AAAMAGFPPEITVTPPTP], and phosphorylated CABIN1-L-1 [Biotin-AAAMAGFPPEITVTPP{pTHR}P]). All the peptides were purified by HPLC and were >95% pure, and their molecular weight and composition were analyzed by mass spectrometry.

### Luciferase reporter assay

To measure the transcriptional activity of NFAT, we cotransfected Jurkat T cells with GST-tagged peptides and NFAT-luc by electroporation. After 24 h, we pretreated the cells with 0.5 μM FK506 for 1 h and then treated them with 40 nM PMA and 1 μM ionomycin for 8 h. For the mammalian two-hybrid assay, we cotransfected HEK293T cells with GAL4 DBD-tagged CABIN1, VIVIT, chimeric and substituted peptides, VP16 activation domain-tagged CNAβ_2_, and pG5-luc (luciferase reporter plasmid containing the GAL4-responsive promoter) for 24 h. We lysed the harvested cells with a luciferase assay lysis buffer (17 mM KH_2_PO_4_, 183 mM K_2_HPO_4_, pH 7.8, 0.2% [v/v] Triton X-100) and sonicated them briefly. We measured luciferase activity for each cell lysate using an Infinite M200 (Tecan, Männedorf, Switzerland). In addition, we confirmed the expression level of the transfected proteins by Western blotting.

### Immunoprecipitation assay

We lysed the cells in IP150 buffer and immunoprecipitated Flag- and HA-tagged proteins with Anti-FLAG ® M2 Affinity Gel (Sigma–Aldrich) and Pierce™ Anti-HA Agarose (Thermo Fisher Scientific), respectively. The composition of the buffer and the subsequent process was described previously^21^. To quantify the western blot results, we measured the band areas using ImageJ (https://imagej.nih.gov/ij/).

### Protein purification and in vitro binding assay

GST-tagged peptides and His-tagged CNAα and β_2_ were expressed in *Escherichia coli* BL21(DE3)pLysS. When the OD_600_ reached 0.5, we treated the bacteria with IPTG (0.1 mM overnight at 18 °C or 1 mM for 4 h at 37 °C). We purified the proteins as described previously^22^. We performed a His-pulldown assay with 0.5 ml of IP150 buffer with 0.5 mM EDTA. We mixed His-tagged CNA with GST-tagged peptides and captured it on Ni-NTA agarose beads (Qiagen, Hilden, Germany). We washed the beads three times with binding buffer and analyzed the proteins by western blotting. We pulled down the GST-tagged peptides using Glutathione Sepharose™ 4 Fast Flow (Cytiva, Marlborough, MA, USA) and the biotin-labeled peptides using Streptavidin Agarose Resin (Thermo Fisher Scientific).

### In vitro kinase assay

We used the purified GST-tagged peptides for the in vitro kinase assays with activated recombinant kinases obtained from Proqinase GmbH (Freiburg, Germany). We performed the kinase assays in reaction buffer (50 mM HEPES-NaOH, pH 7.5, 3 mM MgCl_2_, 3 mM MnCl_2_, 3 μM sodium orthovanadate, 1 mM dithiothreitol, 50 μM unlabeled ATP, 5 μCi ^32^P-γ-ATP) for 1 h at 30 °C. We stopped the reaction by adding 5× sample buffer and separated the proteins using SDS-PAGE. The gel was stained with Coomassie Brilliant Blue, dried, and developed on X-ray film.

### Confocal microscopy and analysis

We transfected Jurkat T cells with the pCAG-EGFP-tNFATC2-IRES-mCherry-peptide by electroporation. After two days, we pretreated these cells with 0.5 μM FK506 for 30 min. Then, we treated them with 40 nM PMA and 1 μM ionomycin under culture conditions for 4 h. Before imaging by a confocal laser scanning microscope (Nikon, Tokyo, Japan), we treated the cells with 10 μg/ml Hoechst 33342 (Invitrogen, Waltham, MA, USA) to stain nuclei. Colocalization analysis of mCherry and Hoechst was performed with ImageJ and its plugin JACoP to measure the Manders coefficient^23,24^.

### Calcineurin activity assay

We measured calcineurin phosphatase activity in vitro using a calcineurin phosphatase assay kit (BML-AK804, Enzo Life Sciences, Farmingdale, NY, USA). We incubated GST or GST-tagged peptides with CNA for 30 min at room temperature and then followed the manufacturer’s instructions. To measure endogenous calcineurin phosphatase activity, we transiently transfected Jurkat T cells with CAG-HA-mCherry-peptides by electroporation. After 24 h, we pretreated the Jurkat T cells with FK506 for 1 h before activating them with PMA and ionomycin. Next, we washed the cells with TBS buffer (20 mM Tris, pH 7.2, 150 mM NaCl), resuspended them with lysis buffer from the calcineurin cellular activity assay kit (BML-AK816, Enzo Life Sciences), and kept them on ice for 30 min. We collected the lysate supernatant and removed free phosphate by gel filtration using the Desalting Column Resin included in the kit. Next, we followed the manufacturer’s instructions.

### Real-time quantitative PCR

We collected Jurkat T cells with QIAzol Lysis Reagent (Qiagen) to extract total RNA. We reverse transcribed cDNAs from isolated RNA using AMV Reverse Transcriptase and random hexamers. We performed real-time quantitative PCR for each cDNA using TB Green® Premix Ex Taq™ (TaKaRa, Kusatsu, Japan) on a CFX Connect Real-time PCR Detection System (Bio-Rad, Hercules, CA, USA) and normalized the expression to that of *18* *S rRNA* or *GAPDH*. Supplementary Table 1 describes the primers used.

### mRNA sequencing and analysis

We pretreated Jurkat T cells expressing HA-mCherry-tagged peptides with FK506 for 1 h before activating them with PMA and ionomycin for 8 h. We then extracted RNA from these cells. Construction of the cDNA library and next-generation sequencing was performed at LAS (Gimpo, Korea). We sequenced all samples on an Illumina NextSeq 500 system with 75 paired-end reads. For the RNA sequencing analysis, reads for each sample were aligned to the human genome (GRCh37/hg19 genome assembly) using STAR 2.4.0.1^25^ with default settings. We used HOMER tools to quantify and normalize the defined genes in RefSeq transcripts. FPKM normalized genes were hierarchically clustered using Cluster3.0^26^. We visualized refined data with Java Treeview (http://jtreeview.sourceforge.net). We conducted gene ontology analysis of each cluster using the DAVID web tool (https://david.ncifcrf.gov). Finally, we visualized the expression levels of target genes using the Integrative Genomics Viewer (http://software.broadinstitute.org/software/igv). The raw data were submitted to the NCBI Gene Expression Omnibus under accession number GSE185561.

### Flow cytometry

We quantified the expression of mCherry-tagged VIVIT peptides using LSRII (SORP) (Becton Dickinson, Franklin Lakes, NJ, USA). We analyzed the results with FlowJo (Tree Star, Inc., Ashland, OR, USA).

### Mass spectrometry

We purified HA-mCherry-tagged CABIN1-L-1-2 peptides expressed in Jurkat T cells by immunoprecipitating 4 mg of lysates with Pierce™ Anti-HA Agarose. We eluted the bead-bound proteins by competition with 1 mg/ml HA peptide. After separation by SDS-PAGE and staining with Coomassie Brilliant Blue, we analyzed the purified proteins by LC-MS/MS (ProteomeTech, Inc., Seoul, Korea).

### Surface plasmon resonance

Using a Biacore T200 (Cytiva), we captured the biotin-labeled peptides on the surface of a Series S Sensor Chip SA (Cytiva). We serially diluted purified His-tagged CNA in HBS-EP + (10 mM HEPES, pH 7.4, 0.15 M NaCl, 3 mM EDTA, 0.05% surfactant P20) and passed it sequentially over three flow cells (flow cell 1: biotin-VEET, flow cell 2: biotin-CABIN1-L-1, flow cell 3: biotin-p-CABIN1-L-1). Each cycle was performed at 25 °C at a flow rate of 30 μl/min with 120 s of contact and 240 s of dissociation. We analyzed the kinetics of interactions using BIAevaluation 3.2 RC1 (Cytiva).

### Statistics

All data are presented as the mean ± standard deviation from at least three biologically independent trials. We calculated *p*-values using a two-tailed Student’s *t-*test to compare two groups and using a two-tailed one-way ANOVA with Tukey’s multiple comparison test to analyze multiple groups. The *p*-values <0.05, <0.01, and <0.001 appear as single, double, and triple asterisks, respectively. NS indicates a nonsignificant difference (*p* ≥ 0.05).

## Results

### The 10 amino acids of CABIN1(2146–2155) are essential for the interaction with calcineurin

**Fig. 1:**
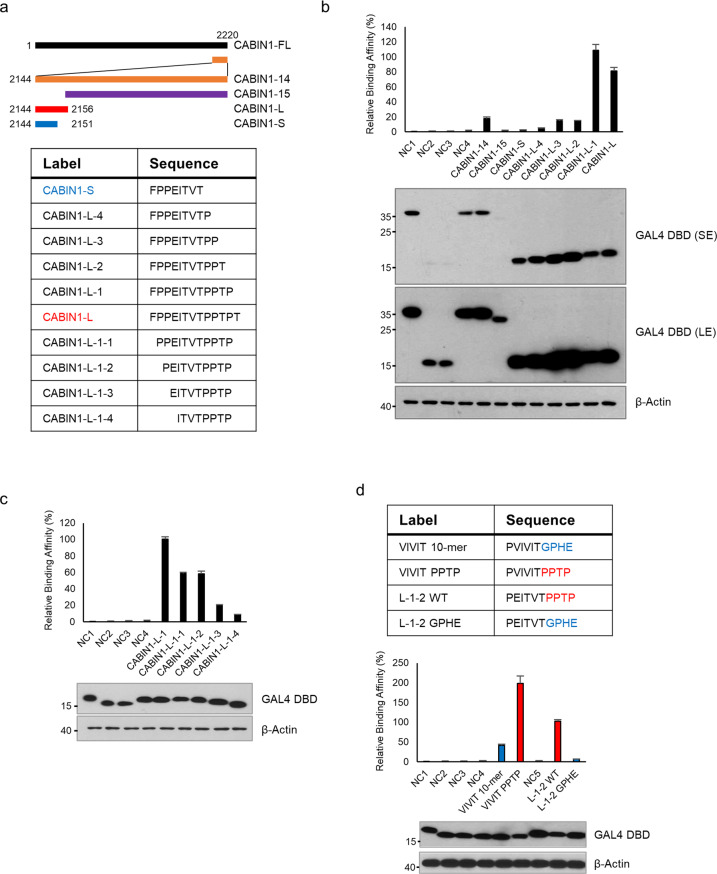
The short peptide from the CABIN1 *C*-terminus is essential for interaction with calcineurin. **a** Schematic diagram and sequence of CABIN1 fragments. **b, c** A mammalian two-hybrid assay was performed using VP16 AD-CNAβ_2_ and GAL4 DBD-CABIN1 peptides (**b** CABIN1-14, -15, -S ~ L; **c** CABIN1-L-1 ~ L-1-4) in HEK293T cells. **d** Sequences of CABIN1-L-1-2, the VIVIT 10-mer and the chimeric peptide (top). A mammalian two-hybrid assay using these peptides was performed (bottom). The compositions of the transfected DNA are described in Supplementary Fig. 1a–c. All experiments were repeated three times and are presented as the mean ± standard deviation. The expression levels of the peptides were confirmed by western blotting. SE short exposure, LE long exposure, NC negative control, WT wild-type.

To determine the minimal sequence binding to calcineurin, we separated the first 13-amino acid segment containing the “PxIxIT” motif (named CABIN1-L) from CABIN1-14 (Fig. 1a). We performed a mammalian two-hybrid assay using the GAL4 DBD-tagged CABIN1 peptides and VP16 activation domain-tagged CNA (Fig. 1b, c, Supplementary Fig. 1a, b). Our results confirmed that CABIN1-14 binds to CNA, as previously reported^20^, while the other sequences (CABIN1-15) did not.

We designed several peptides by sequentially removing the CABIN1-L *C*-terminal amino acid residues (Fig. 1a; CABIN1-S–CABIN1-L). The mammalian two-hybrid interaction assay showed that CABIN1-L and CABIN1-L-1 had a significantly stronger affinity for CNA than CABIN1-14. However, CABIN1-L-2 completely lost its binding affinity to CNA despite its higher expression level (Fig. 1b). Next, we designed additional peptides by sequentially removing the CABIN1-L-1 *N*-terminal amino acid residues (Fig. 1a; CABIN1-L-1-1–CABIN1-L-1-4). CABIN1-L-1-1 and CABIN1-L-1-2 still bound to CNA, but CABIN1-L-1-3 lost its binding affinity (Fig. 1c). The immunoprecipitation assays using the GAL4 DBD-tagged CABIN1 peptide and Flag-tagged CNA showed identical results (Supplementary Fig. 1d, e). These results demonstrate that the 10 amino acids from proline 2146 to proline 2155 of full-length CABIN1 form the minimal sequence needed for interaction with calcineurin.

### The PPTP sequence following the “PxIxIT” motif is critical for the interaction with calcineurin

Previous research demonstrated the biochemical and structural importance of the “PxIxIT” motif^18,27^, but the role of the neighboring sequences around the “PxIxIT” motif in calcineurin binding remains unclear. Since proline 2155 does not belong to the “PxIxIT” motif, we needed to clarify the role of the *C*-terminal “PPTP” sequence following the “PxIxIT” motif. To compare the CABIN1 and VIVIT peptides under the same conditions, we used the VIVIT decamer (VIVIT 10-mer) as a control. We constructed a chimeric peptide by swapping the *C*-terminal sequences of the VIVIT and CABIN1 peptides and conducted a mammalian two-hybrid assay (Fig. 1d, Supplementary Fig. 1c). The *C*-terminal “PPTP” sequence enhanced the interaction with CNA by 4.76-fold (VIVIT PPTP vs. VIVIT 10-mer), while the *N*-terminal “PVIVIT” enhanced it by 1.93-fold (VIVIT PPTP vs. L-1-2 WT). This result suggests that the *C*-terminal “PPTP” sequence also plays an important role in the interaction with calcineurin.

Next, we investigated whether the *N*-terminal sequence in front of “PxIxIT” (“AGPH” in the VIVIT peptide and “FP” in CABIN1-L-1) contributed to the interaction with calcineurin. Adding “AG,” “PH,” or “FP” before the “PxIxIT” motif did not affect the interaction with calcineurin (Supplementary Fig. 2a, b). In addition, these sequences did not affect the inhibitory effect of the calcineurin-NFAT pathway (Supplementary Fig. 2c). In the case of the VIVIT peptide, the *C*-terminal glutamate repeats decreased protein stability (Supplementary Fig. 3a–d).

### The CABIN1 peptide is a stronger calcineurin-NFAT pathway inhibitor than the VIVIT peptide

**Fig. 2:**
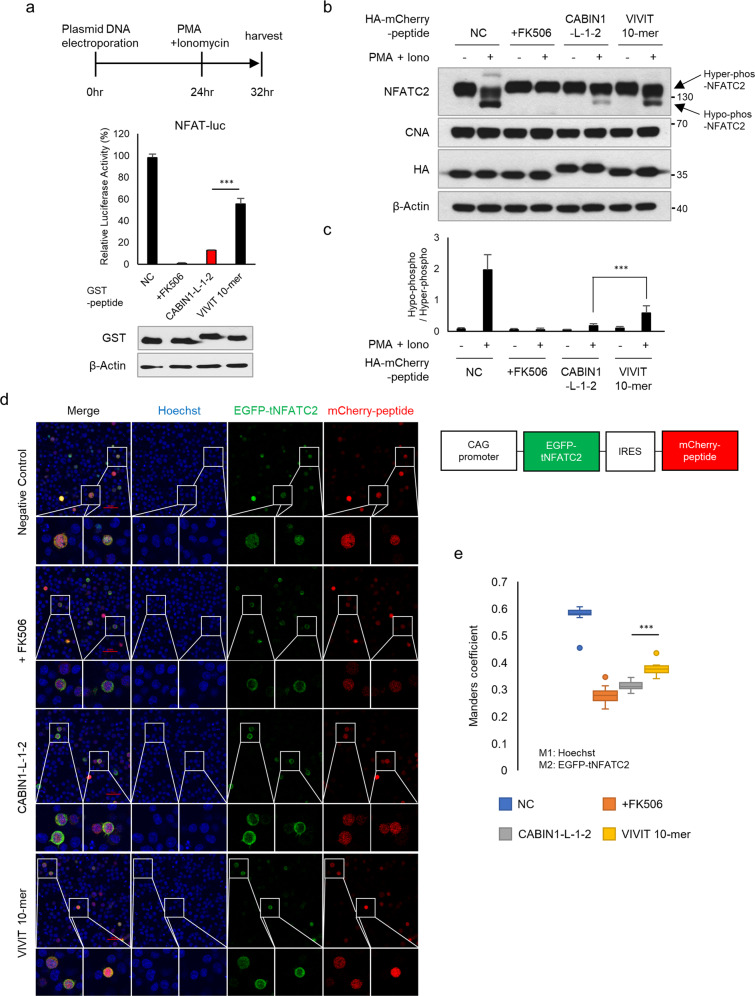
The CABIN1 peptide inhibits the CN-NFAT pathway more efficiently than the VIVIT peptide. **a** A luciferase reporter assay was performed to measure NFAT transcriptional activity. Jurkat T cells expressing GST-tagged peptides were treated with PMA and ionomycin for activation. Jurkat T cells expressing only GST were used as a negative control, and cells treated with 0.5 μM FK506 were used as a positive control (*n* = 3). **b** NFAT mobility shifting assays were performed using Jurkat T cells expressing HA-mCherry-tagged peptides. For dephosphorylation of NFATC2, Jurkat T cells were treated with PMA and ionomycin for 2 h. **c** Hyper- and hypophosphorylated NFATC2 of **b** was measured using ImageJ, and the hypo/hyper ratio was calculated (*n* = 7). **d** Snapshot images of the activated Jurkat T cells expressing the indicated peptides and tNFATC2 were obtained using confocal microscopy. The expression and localization of peptides and tNFATC2 are shown as mCherry (red) and EGFP (green), respectively. Nuclei were stained with Hoechst (blue). Scale bar, 50 μm **e** Nuclear-localized tNFATC2 was measured by calculating the colocalization ratio between Hoechst (M1) and EGFP (M2). (NC and FK506, *n* = 10; CABIN1-L-1-2 and VIVIT 10-mer, *n* = 12) Data are representative of independently repeated experiments and presented as the mean ± standard deviation. Statistical differences were determined using a two-tailed Student’s *t*-test (**a, c**) or one-way ANOVA (**e**). ***p < 0.001, NC negative control.

To investigate the inhibitory effect of the CABIN1 peptide on the calcineurin-NFAT pathway, we cotransfected the GST-tagged CABIN1-L-1-2 or VIVIT peptide into Jurkat T cells with a luciferase reporter gene under the control of three repeated NFAT binding sites. Upon treatment with PMA and ionomycin, expression of the GST-tagged CABIN1-L-1-2 or VIVIT peptide repressed luciferase activity compared with the negative control. The inhibitory effect of CABIN1-L-1-2 was approximately four times stronger than that of the VIVIT peptide (Fig. 2a). To confirm that this result is due to NFATC2 dephosphorylation by calcineurin, we performed an NFAT mobility shifting assay. In T cells, NFATC1 and NFATC2 are hyperphosphorylated, and calcineurin dephosphorylates them in response to calcium signaling^8^. Hyperphosphorylated NFATs are localized in the cytosol, whereas dephosphorylated proteins are localized in the nucleus and migrate faster in gels than hyperphosphorylated proteins^28^. Similar to FK506, the CABIN1 peptide significantly inhibited the NFATC2 band shift upon treatment with PMA and ionomycin (Fig. 2b). The VIVIT peptide also reduced the dephosphorylation of NFATC2, but its inhibitory effect was weaker than that of the CABIN1 peptide (Fig. 2b, c).

Next, we constructed plasmids expressing EGFP-tagged truncated NFATC2 (tNFATC2; 2–460 amino acids) and mCherry-tagged each peptide to confirm the contribution of these peptides to NFAT nuclear import. The proportion of nuclear-localized tNFATC2 was lowest in the FK506-treated cells and slightly higher in the Jurkat T cells coexpressing the CABIN1-L-1-2 peptide. Moreover, the Jurkat T cells coexpressing the VIVIT peptide showed a significantly higher proportion of nuclear-localized tNFATC2 than the cells coexpressing the CABIN1-L-1-2 peptide (Fig. 2d, e). Altogether, these results demonstrate that the CABIN1 peptide blocks the dephosphorylation and nuclear translocation of NFATC2 more efficiently than the VIVIT peptide does.

### The CABIN1 peptide blocks T lymphocyte activation

**Fig. 3:**
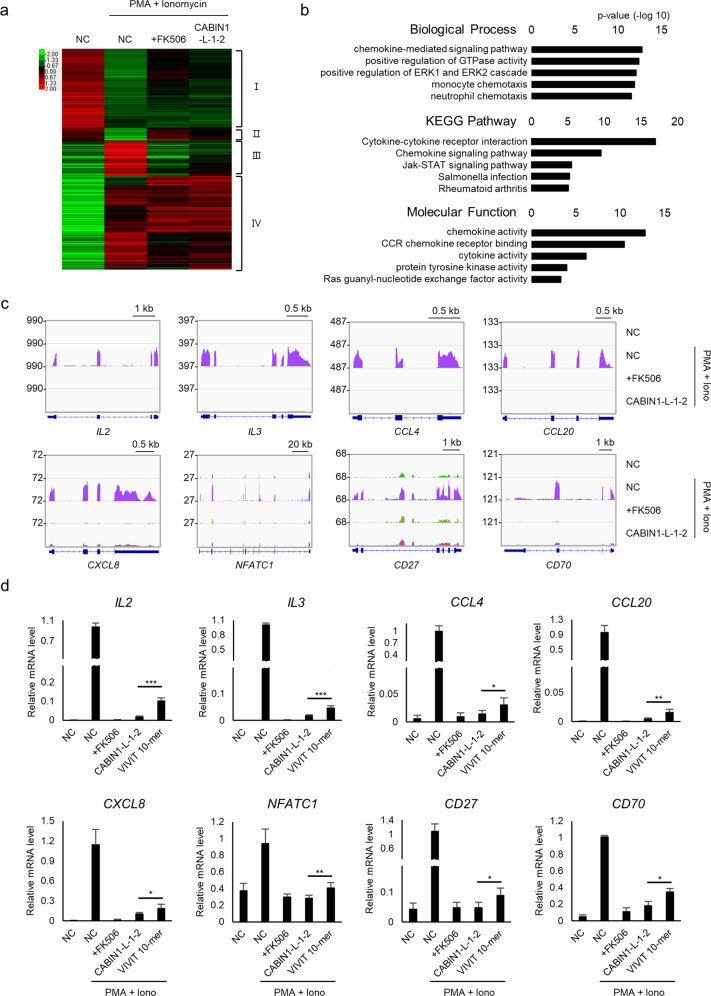
The CABIN1 peptide inhibits T-cell activation. **a** Heatmap of altered genes affected by FK506 treatment or CABIN1 peptide expression upon T-cell activation. **b** Gene Ontology analysis of cluster III. **c** mRNA expression levels of genes included in cluster III. **d** qRT-PCR data of genes in **c**. The experiment was independently repeated at least three times, and the data are presented as the mean ± standard deviation. Statistical differences were determined using two-tailed Student’s *t-*test. *p < 0.05, **p < 0.01, ***p < 0.001, NC negative control.

To confirm whether the CABIN1 peptide blocks T lymphocyte activation by interfering with the calcineurin-NFAT interaction, we sequenced the RNA of Jurkat T-cell lines stably expressing the negative control (mCherry only) and mCherry-tagged CABIN1 peptide. We found 685 genes affected by PMA and ionomycin treatment in the negative control cells (Fig. 3a, Supplementary Table 2). We focused on cluster III of the four clusters, which contained genes with increased expression levels in the negative control group upon treatment with PMA and ionomycin and reduced expression levels in the CABIN1-expressing cells and the FK506-treated cells (Supplementary Table 3). Gene Ontology analysis revealed that the genes in cluster III are related to chemokines, cytokine signaling, and the immune response (Fig. 3b). Furthermore, upon T-cell activation, the CABIN1 peptide, and FK506 reduced the RNA levels of representative T-cell activation markers, including *IL2*, *IL3*, *NFATC1*, and various chemokine ligands (*CCL4*, *CCL20*, and *CXCL8*). In addition, the CABIN1 peptide and FK506 repressed *CD27* and *CD70*, which regulate the T-cell costimulatory pathway (Fig. 3c). Real-time quantitative PCR analysis of these genes showed that the CABIN1 peptide reduced the RNA levels of these genes more efficiently than the VIVIT peptide (Fig. 3d).

### The CABIN1 peptide does not inhibit calcineurin phosphatase activity

**Fig. 4:**
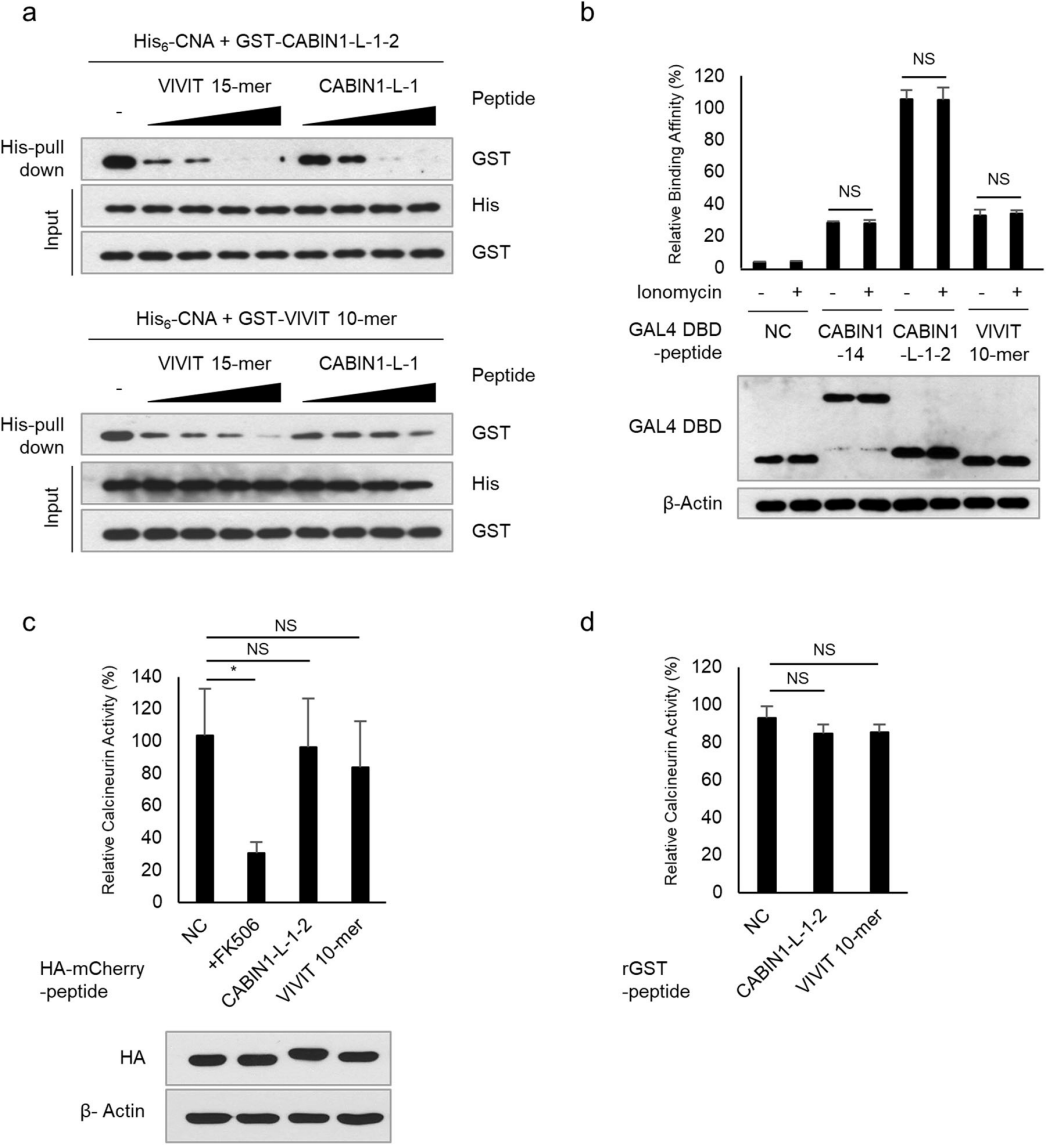
The CABIN1 peptide does not affect calcineurin’s phosphatase activity. **a** An in vitro peptide competition assay was conducted with the VIVIT 15-mer and CABIN1-L-1 peptides in a dose-dependent manner. The molar ratios of the GST-tagged peptide to the competing peptide ranged from 1:50 to 1:400. **b** A mammalian two-hybrid assay was performed using VP16 AD-CNAβ_2_, and GAL4 DBD-peptides in the HEK293T cells with or without 1 μM ionomycin treatment. pM empty vector was used as a negative control. **c** Calcineurin cellular activity assays were performed using Jurkat T cells expressing HA-mCherry-tagged peptides. Jurkat T cells expressing only HA-mCherry were used as a negative control, and cells treated with FK506 were used as a positive control. **d** In vitro calcineurin phosphatase activity assays using purified GST-tagged peptides. Purified GST was used as a negative control. All experiments were repeated three times, and the data are presented as the mean ± standard deviation. Statistical differences were determined using one-way ANOVA. *p < 0.05, NS nonsignificant, NC negative control.

The VIVIT peptide binds to distinct regions of the CNA catalytic site and disrupts the CNA-NFAT interaction^27^. To confirm that the CABIN1 and VIVIT peptides have the same mechanism of action, we conducted a peptide competition assay. The CABIN1 peptide dose-dependently interfered with the interaction between the GST-tagged VIVIT peptide and His_6_-tagged CNA and vice versa (Fig. 4a). In addition, ionomycin treatment did not affect the interaction between CNA and the CABIN1 or VIVIT peptide (Fig. 4b). This result confirms that the CABIN1 peptide, VIVIT peptide, and fused tag (GAL4 DBD) do not interfere with the active site of CNA because CNA’s autoinhibitory domain blocks the active site in the steady-state and releases it through a calcium-induced conformational change upon ionomycin treatment^5^. Next, we measured calcineurin phosphatase activity in Jurkat T cells expressing each peptide or treated with FK506 (Fig. 4c). FK506, which interferes with the active site of CNA, decreased the phosphatase activity of CNA, but the CABIN1 and VIVIT peptides did not. These peptides also did not reduce the phosphatase activity of CNA in vitro (Fig. 4d).

### The *C*-terminal PPTP sequence is important for interaction with calcineurin

**Fig. 5:**
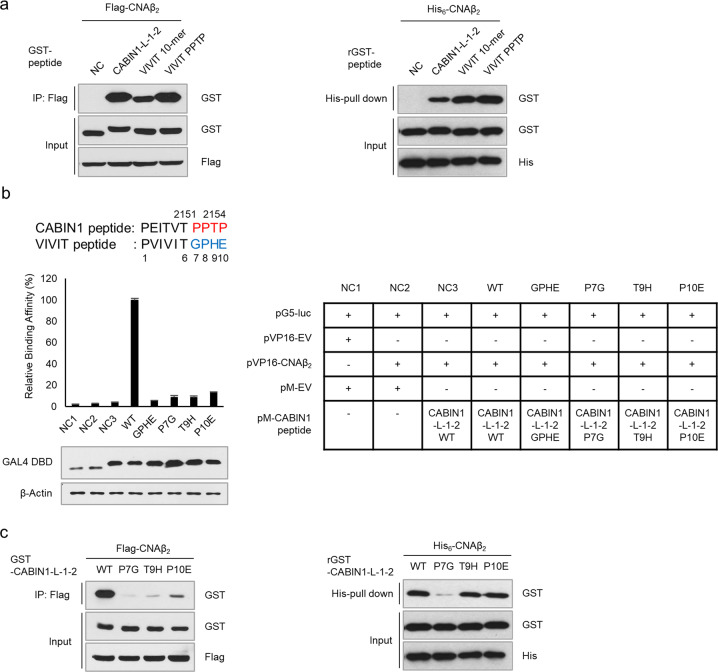
The PPTP sequence is critical for the interaction with calcineurin at the cellular level. **a** Immunoprecipitation assay in HEK293T cells (left) and an in vitro His-pulldown assay (right) using the indicated tagged CNAβ_2_ and peptides. **b** A mammalian two-hybrid assay was conducted using CNAβ_2_ and substituted CABIN1 peptides to verify binding affinity (*n* = 3). HEK293T cells were transfected as described in the right panel. **c** Immunoprecipitation assay in HEK293T cells (left) and an in vitro His-pulldown assay using the indicated tagged CNAβ_2_ and wild-type or substituted CABIN1 peptides (right). Values shown are the mean ± standard deviation. NC negative control, WT wild-type.

To understand how the “PPTP” sequence contributes to the binding affinity to CNA, we compared the binding affinity of each GST-tagged peptide for CNA at the cellular level and in vitro (Fig. 5a). The “PPTP” sequence enhanced CNA binding affinity only at the cellular level, not in vitro, suggesting that this effect was due to post-translational modifications (PTMs) on residues of “PPTP.” Next, we substituted one or all of the PPTP residues with amino acids corresponding to each residue of the GPHE sequence of the VIVIT peptide. We conducted a mammalian two-hybrid assay using these constructs and CNA to determine which modification affected the affinity (Fig. 5b). As a result, all the substitutions dramatically disrupted the interaction with CNA, implying that every residue in the “PPTP” sequence is crucial for the interaction with CNA. This result suggests that the “PPTP” sequence could be a substrate motif of an upstream enzyme capable of modifying residues. Subsequent experiments using these substitutions revealed that threonine 9 and proline 10 of the CABIN1 peptide might be targets of PTM because, in vitro, peptides with substitutions at these residues exhibited binding affinities for CNA comparable to that of wild-type CABIN1, while in cellular experiments, all the substitutions disrupted the interaction with calcineurin (Fig. 5c).

### Phosphorylation of CABIN1 T2154 is critical for the interaction with calcineurin

We investigated the phosphorylation and *O*-linked glycosylation of threonine residues and hydroxylation of proline residues of the peptides containing “PPTP”.﻿ First, we investigated whether proline residues of “PPTP” were hydroxylated. We treated Jurkat T cells expressing the mCherry-tagged CABIN1 peptide with dimethyloxalylglycine (DMOG), a prolyl hydroxylase inhibitor (Supplementary Fig. 4a, b). While DMOG stabilized hypoxia-inducible factor-1alpha (HIF-1α), it did not affect the CABIN1 peptide and CNA interaction. Furthermore, we detected no hydroxyproline in the GST-tagged CABIN1 peptide or VIVIT peptide (Supplementary Fig. 4c). Next, we assessed *O*-linked glycosylation at the threonine residue of “PPTP.” The *O*-linked glycosylation of GST-tagged peptides containing “PPTP” (CABIN1-L-1-2 and VIVIT PPTP) was not higher than that of the negative control or VIVIT peptide, indicating that no *O*-linked glycosylation occurred in the “PPTP” sequence (Supplementary Fig. 4c).

**Fig. 6:**
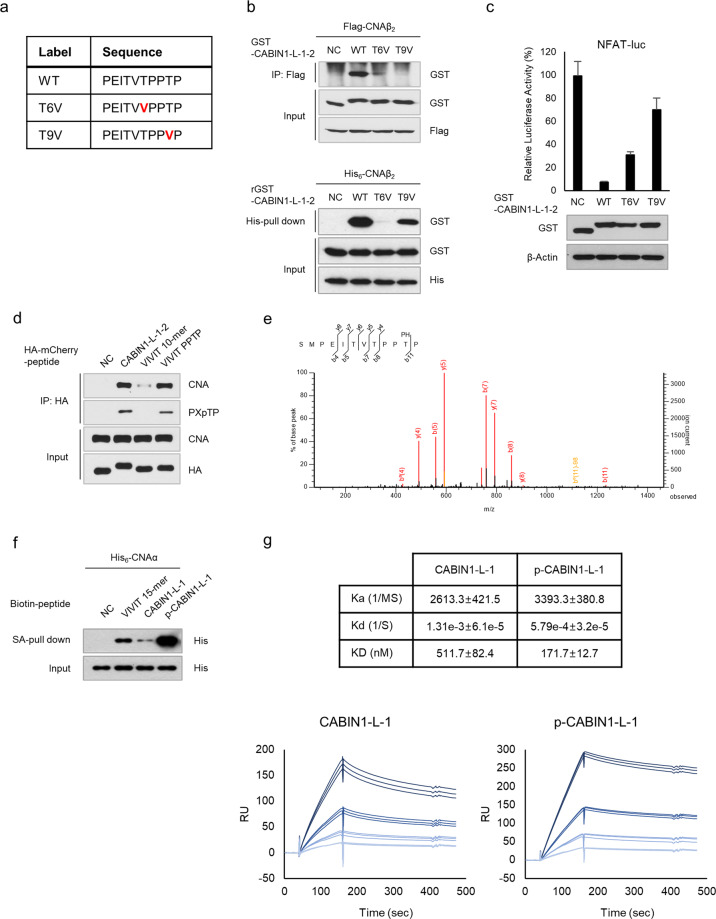
Phosphorylation of CABIN1 T2154 is important for binding to calcineurin. **a** Sequence of threonine- to valine-substituted (TV) CABIN1 peptides. **b** Immunoprecipitation assay in HEK293T cells (top) and an in vitro His-pulldown assay (bottom) using the indicated tagged CNAβ_2_ and wild-type or TV CABIN1 peptides. **c** A luciferase reporter assay was performed to measure NFAT transcriptional activity in the Jurkat T cells expressing GST-tagged CABIN1 peptides under PMA and ionomycin treatment (*n* = 3). **d** Immunoprecipitation assay of the Jurkat T cells expressing HA-mCherry-tagged peptides using HA antibody. Peptide-bound CNA and phosphorylated threonine were detected by Western blotting. **e** Phosphorylation of the 9th threonine of the CABIN1 peptide (threonine 2154 of the full-length CABIN1) in Jurkat T cells was detected by LC-MS/MS. **f** An in vitro pulldown assay was performed using streptavidin-conjugated agarose beads. Biotinylated peptide-bound CNA was detected with His antibody. VEET peptide was used as a negative control. **g** CNA binding to biotinylated CABIN1 peptides with or without 9th threonine phosphorylation was measured by SPR. CNA concentrations ranged from 1.25, 2.5, 5, and 10 nM. The kinetics of interactions were calculated using the data of the CABIN1 peptide-captured cells minus the negative control peptide-captured cell. Values shown are the mean ± standard deviation. RU response units, NC negative control.

Previous phosphoproteomics experiments demonstrated the phosphorylation of CABIN1 threonines 2151 and 2154 during mitosis^29^. To identify the modified residue in our model, we substituted each threonine with valine, which bears a methyl group instead of the hydroxyl group of threonine (Fig. 6a). Substituting threonine 6 (threonine 2151 of the full-length CABIN1) with valine (T6V) abolished the interaction between the CABIN1 peptide and CNA in the cellular and in vitro experiments. In contrast, substituting threonine 9 (threonine 2154 of the full-length CABIN1) with valine (T9V) abolished the interaction in the cellular experiment but not in vitro (Fig. 6b). Moreover, the T9V substitution restored the CABIN1-suppressed transcriptional activity of NFAT more efficiently than the T6V substitution (Fig. 6c). These results suggest that threonine 9 undergoes PTM, which increases the inhibitory effect on the CNA-NFAT pathway.

Indeed, we detected threonine 9 phosphorylation by Western blots using an antibody against phospho-MAPK substrates (PXpTP) in the Jurkat T cells expressing mCherry-tagged peptides (Fig. 6d). To directly detect the phosphorylated CABIN1 peptide in cells, we immunoprecipitated the HA-mCherry-tagged CABIN1 peptide with an anti-HA antibody from the Jurkat T cells expressing the protein. We analyzed the purified protein with LC-MS/MS and observed threonine 9 phosphorylation (Fig. 6e). Next, we synthesized a biotin-labeled VIVIT peptide, CABIN1 peptide, and phosphorylated CABIN1 peptide. A streptavidin pulldown assay with these peptides showed that CABIN1 phosphorylation dramatically increased the affinity for CNA (Fig. 6f). Finally, we quantitatively analyzed the binding affinity through surface plasma resonance (SPR). We observed that phosphorylation of the 9th threonine of the CABIN1 peptide increased the binding affinity by approximately threefold (Fig. 6g). Collectively, these results demonstrate that the CABIN1 peptide is a more efficient in vivo inhibitor than the VIVIT peptide because the phosphorylation of the threonine in “PPTP” enhances the binding affinity for CNA.

### p38MAPK phosphorylates threonine 9 of the CABIN1 peptide

**Fig. 7:**
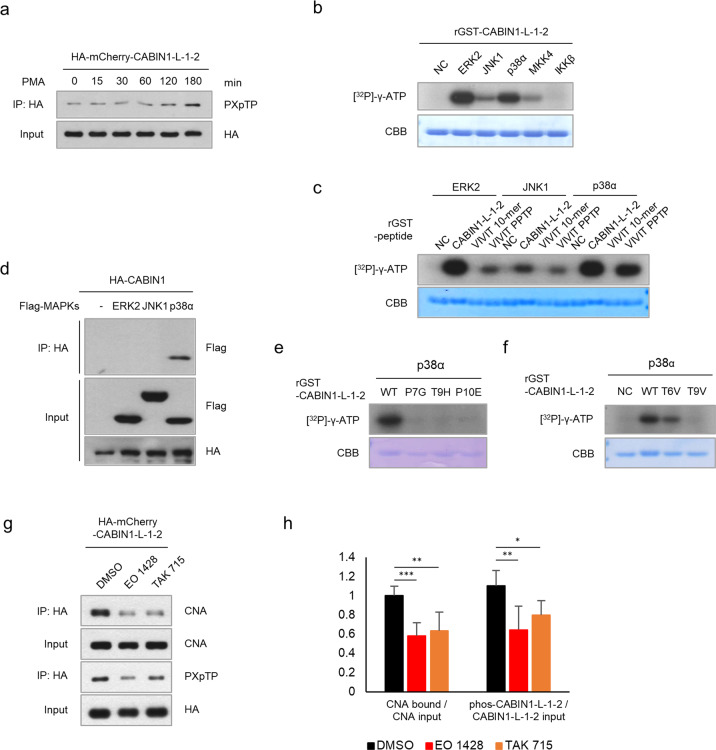
p38MAPK phosphorylates the 9th threonine of the CABIN1 peptide. **a** Jurkat T cells expressing HA-mCherry-tagged CABIN1 peptide were treated with 40 nM PMA in a time-dependent manner. The phosphorylated threonine of the immunoprecipitated peptide with HA antibody was detected with [PXpTP] antibody. **b** In vitro kinase assays using purified GST-tagged CABIN1 peptide and PKC downstream kinases. **c** In vitro kinase assays using purified GST-tagged peptides and kinases included in the MAPK family. **d** Immunoprecipitation assays using Flag-MAPKs and HA-CABIN1 in HEK293T cells. **e, f** In vitro kinase assays using p38α and substituted CABIN1 peptides. **g** Jurkat T cells expressing HA-mCherry-tagged CABIN1 peptide were treated with 10 μM p38α inhibitors for 16 h. Immunoprecipitation was performed with HA antibody and detected by western blotting. **h** Phosphorylated threonine and peptide-bound CNA of **g** were measured using ImageJ, and the ratio of phosphorylation and calcineurin binding to the CABIN1 peptide was calculated (*n* = 6). Values shown are the mean ± standard deviation. Statistical differences were determined using one-way ANOVA. *p < 0.05, **p < 0.01, ***p < 0.001, CBB Coomassie Brilliant Blue, NC negative control.

PMA, an activator of PKC, induces CABIN1 hyperphosphorylation, but PKC does not directly phosphorylate CABIN1^20^. Therefore, we investigated whether PMA causes the phosphorylation of the CABIN1 peptide—specifically the threonine of “PPTP”—and through which kinase. After PMA treatment, CABIN1 peptide phosphorylation increased two hours after treatment (Fig. 7a). To identify kinases that can directly phosphorylate the CABIN1 peptide, we performed an in vitro phosphorylation assay with kinase candidates activated through PKC. We found that the MAPK family (EKR, JNK, and p38) and MKK4 directly phosphorylate the CABIN1 peptide (Fig. 7b). We focused on the MAPKs, considering that “PPTP” is a perfectly suited substrate motif for this family. ERK2, JNK1, and p38α phosphorylated the purified recombinant GST-tagged peptides containing the “PPTP” sequence (CABIN1-L-1-2 and VIVIT PPTP) but not the GST-tagged VIVIT peptide (Fig. 7c). To investigate which kinases interact with CABIN1 in nature, we conducted an immunoprecipitation assay. This assay revealed that only p38α was bound to CABIN1 at the cellular level (Fig. 7d). In addition, p38α did not phosphorylate the substituted PPTP sequences in vitro (Fig. 7e, f). We then confirmed that selective p38 inhibitors, such as EO 1428 or TAK 715, significantly reduced phosphorylation of the CABIN1 peptide, thereby decreasing its binding affinity for CNA (Fig. 7g, h). These results suggest that p38α is an upstream kinase of the CABIN1 peptide in nature.

## Discussion

FK506 and cyclosporin A, respectively cooperating with FKBP12 or cyclophilin, spatially hinder the substrates’ access to the active site of calcineurin, inhibiting its phosphatase activity^30,31^. Long-term treatment with these drugs increases the expression of TGF-β and profibrogenic genes, such as collagen, fibronectin, and metalloproteinases^32^. Consequently, FK506 and cyclosporin A cause severe side effects, such as nephrotoxicity, fibrogenesis, neurotoxicity, and diabetes^17^.

To overcome these side effects, researchers have developed alternative inhibitors that specifically perturb the calcineurin-NFAT pathway. Among them, the VIVIT peptide showed better results than FK506 on allogeneic islet transplantation in mice without reducing insulin secretion^33^. Furthermore, the VIVIT peptide showed potential as an alternative calcineurin inhibitor in treating cardiac hypertrophy and restenosis^34,35^. This peptide also successfully suppressed the development of microbiota-dependent colorectal cancer by inhibiting the calcineurin-NFAT pathway in intestinal epithelial cells^36^.

However, some characteristics of the VIVIT peptide (such as its stability and affinity) and peptide drug delivery systems require improvement before clinical use^19^. At present, a few studies have reported attempts to improve the VIVIT peptide. Most of them are related to delivery systems, such as conjugation with various cell-penetrating peptides or the use of viral delivery systems^33,36–38^. However, reports on VIVIT peptide binding affinity improvement are rare. In 2014, Qian, Dougherty et al. reported that the artificial peptidyl inhibitor ZIZIT-cisPro had a better binding affinity than the VIVIT peptide^39^. However, since ZIZIT-cisPro does not exist in nature, it could trigger an immune response.

This study identified a key peptide sequence in the *C*-terminal fragment of CABIN1 that inhibited the calcineurin-NFAT pathway and clarified its mechanism. Based on this study, the CABIN1 peptide has two advantages over the VIVIT peptide. First, the CABIN1 peptide has a higher binding affinity for calcineurin than the VIVIT peptide. The CABIN1 peptide contains an *N*-terminal “PxIxIT” motif and a “PPTP” sequence, which is different from the *C*-terminal “GPHEE” sequence of the VIVIT peptide. The *C*-terminal “PPTP” sequence endows this peptide with a higher affinity for CNA than the VIVIT peptide (Fig. 1d). This higher affinity for calcineurin allows the CABIN1 peptide to better disrupt the interaction between calcineurin and NFAT, ultimately blocking NFAT dephosphorylation and nuclear translocation more efficiently (Fig. 2b–e). Thus, the CABIN1 peptide successfully suppressed the expression of NFAT target genes upon T-cell activation much more efficiently than the VIVIT peptide (Fig. 3d). These results suggest that the CABIN1 peptide is a more efficient calcineurin inhibitor than the VIVIT peptide. However, the CABIN1 peptide only had a higher affinity at the cellular level, not in vitro (Fig. 5a), indicating that the CABIN1 peptide undergoes PTM which affects its binding affinity. Recent studies have suggested the importance of phosphorylated serine or threonine in calcineurin inhibitory peptides^40,41^. However, no one has directly demonstrated their existence or role in nature. Here, we directly revealed the phosphorylation of the threonine of the “PPTP” sequence (T9) via LC-MS/MS and quantitatively demonstrated that it increases the binding affinity to calcineurin (Fig. 6e, g). During Jurkat T-cell activation, the phosphorylation of nuclear NFAT and CABIN1 peptides by p38MAPK may cooperatively attenuate the immune response by inhibiting the reimportation of phosphorylated NFAT into the nucleus.

Next, the CABIN1 peptide is more stable than the full-length VIVIT peptide (15-mer) under physiological conditions. While comparing the VIVIT 15-mer with the VIVIT decamer, we found an interesting feature of the VIVIT peptide. The mCherry-tagged VIVIT peptide was dramatically less stable with *C*-terminal glutamate repeats (Supplementary Fig. 3b, c). The *C*-terminal glutamine repeat is the *C*-terminal degron targeted by the CUL4/DCAF12 ubiquitin ligase complex^42^. Accordingly, when using the full-length VIVIT peptide, the tag type or location should be considered.

As an alternative calcineurin inhibitor, the CABIN1 peptide may exhibit advantages similar to those of the VIVIT peptide. These peptides compete to interact with CNA, implying that they bind to the same region and have the same mode of action (Fig. 4a). Since the CABIN1 peptide does not affect calcineurin’s phosphatase activity (Fig. 4c, d), it probably does not perturb the dephosphorylation of calcineurin substrates other than NFAT. For instance, KSR2, another calcineurin substrate that interacts with calcineurin via the “LxVP” motif, is dephosphorylated by calcineurin in response to glucose stimulation. Dephosphorylated KSR2 induces ERK activation and insulin secretion^3^. Given that the CABIN1 and VIVIT peptides only carry the “PxIxIT” motif and not the “LxVP” motif, they probably do not interfere with the interaction between KSR2 and calcineurin. A previous report showed that the VIVIT peptide did not decrease insulin secretion in allogeneic islet transplantation in mice^33^, supporting this assumption.

The present study found putative calcineurin target genes, such as *NINJ1* and *ZP4*, that are not regulated via the calcineurin-NFAT pathway. Upon T-cell activation, these genes were repressed by FK506 but unaffected by the CABIN1 and VIVIT peptides (Supplementary Fig. 5a, b). *NINJ1* encodes Ninjurin-1, a transmembrane adhesion molecule involved in axonal growth, inflammation, and cell death^43,44^. Ninjurin-1 regulates inflammatory activation by modulating Toll-like receptor 4 signaling through direct binding to lipopolysaccharides in macrophages^45^. ZP4, a component of the zona pellucida glycoproteins, promotes T-cell proliferation^46^. Further studies should confirm whether these genes are associated with the side effects of FK506.

The interactive mechanism between calcineurin and its binding partners has not been fully elucidated. In addition to CABIN1, other calcineurin binding partners, such as AKAP79, RCAN1, and TRESK, carry a “PxIxIT” motif^5^. However, we found no reports investigating potential PTMs for their calcineurin binding regions. Our previous study showed that an internal fragment of CABIN1 that does not contain the “PxIxIT” or “LxVP” motif also inhibits calcineurin^47^. Here, we revealed the detailed mechanism of CABIN1 as a calcineurin inhibitor and proved the superiority of the CABIN1 peptide over the VIVIT peptide for the first time. Modulation of the inhibitory effect through phosphorylation can be an advantage. Phosphorylation acts as a switch for the CABIN1 peptide, allowing it to target cells or tissues where p38 is highly activated. Given that the NFAT family contributes to the progression and metastasis of many cancers^48^, the CABIN1 peptide could be used as an anticancer agent. Finally, our findings provide an alternative therapeutic strategy against various diseases related to the calcineurin-NFAT pathway.

## Supplementary information

Supplementary information
