# Supplementary material for: The optimized core peptide derived from CABIN1 efficiently inhibits calcineurin-mediated T-cell activation
Source: Exp Mol Med. 2022 May 12;54(5):613–25. doi: 10.1038/s12276-022-00772-6 (PMC9166766; doi:10.1038/s12276-022-00772-6)
Supplement: Supplementary file 1 — Supplementary information [file 12276_2022_772_MOESM1_ESM.pdf]

Supplementary Information

Contents

1. Supplementary Figures

2. Supplementary Tables

1. Supplementary Figures

a

	NC1	NC2	NC3	NC4	CABIN1-14	CABIN1-15	CABIN1-S	CABIN1-L-4	CABIN1-L-3	CABIN1-L-2	CABIN1-L-1	CABIN1-L
pG5-luc	-	+	+	+	+	+	+	+	+	+	+	+
pVP16-EV	-	+	-	+	-	-	-	-	-	-	-	-
pVP16-CNAβ2	+	-	+	-	+	+	+	+	+	+	+	+
pM-EV	-	+	+	-	-	-	-	-	-	-	-	-
pM-CABIN1 Fragment	14	-	-	14	14	15	S	L-4	L-3	L-2	L-1	L

b

	NC1	NC2	NC3	NC4	CABIN1-L-1	CABIN1-L-1-1	CABIN1-L-1-2	CABIN1-L-1-3	CABIN1-L-1-4
pG5-luc	-	+	+	+	+	+	+	+	+
pVP16-EV	-	+	-	+	-	-	-	-	-
pVP16-CNAβ2	+	-	+	-	+	+	+	+	+
pM-EV	-	+	+	-	-	-	-	-	-
pM-CABIN1 Fragment	L-1	-	-	L-1	L-1	L-1-1	L-1-2	L-1-3	L-1-4

c

	NC1	NC2	NC3	NC4	VIVIT 10-mer	VIVIT PPTP	NC5	L-1-2 WT	L-1-2 GPHE
pG5-luc	-	+	+	+	+	+	+	+	+
pVP16-EV	-	+	-	+	-	-	+	-	-
pVP16-CNAβ2	+	-	+	-	+	+	-	+	+
pM-EV	-	+	+	-	-	-	-	-	-
pM-peptide	CABIN1 -L-1-2	-	-	VIVIT 10-mer	VIVIT 10-mer	VIVIT PPTP	CABIN1 -L-1-2	CABIN1 -L-1-2	L-1-2 GPHE

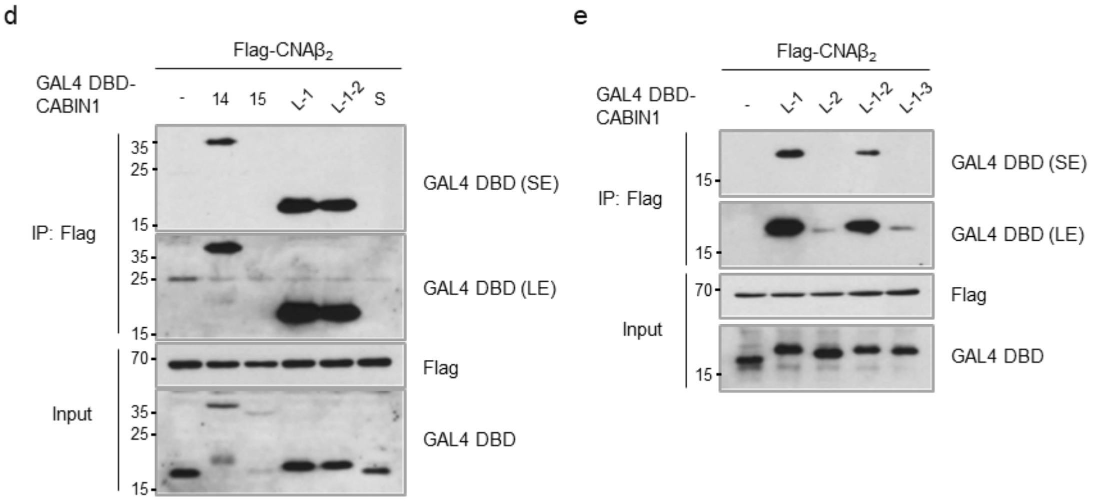

Supplementary Fig. 1 Supporting data for interactions between CNA and CABIN1 fragments a-c Compositions of transfected DNA for mammalian two-hybrid assays in Fig. 1b-

d, respectively. **d, e** HEK293T cells were transfected with Flag-CNAβ_2 and GAL4 DBD-CABIN1 fragments, and immunoprecipitated with Flag antibody. The interaction between CNAβ_2 and each CABIN1 fragment was detected with indicated antibodies. SE, short exposure, LE, long exposure, NC, negative control, WT, wild-type.

a

Label	Sequence
#1	PEITVTPPTP
#2	PVIVITGPHE
#3	PVIVITPPTP
#4	AG FP PEIVTVPPTP
#5	FP PEITVTPPTP
#6	AG FP PVIVITPPTP
#7	AG PH PVIVITGPHEE
#8	AG PH PVIVITPPTP

b

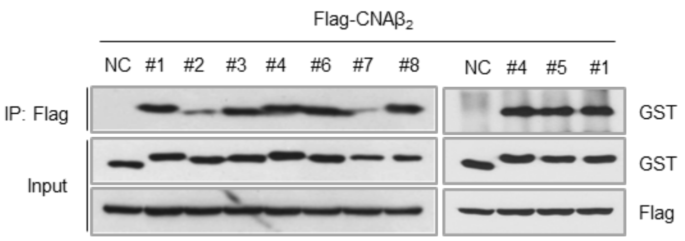

c

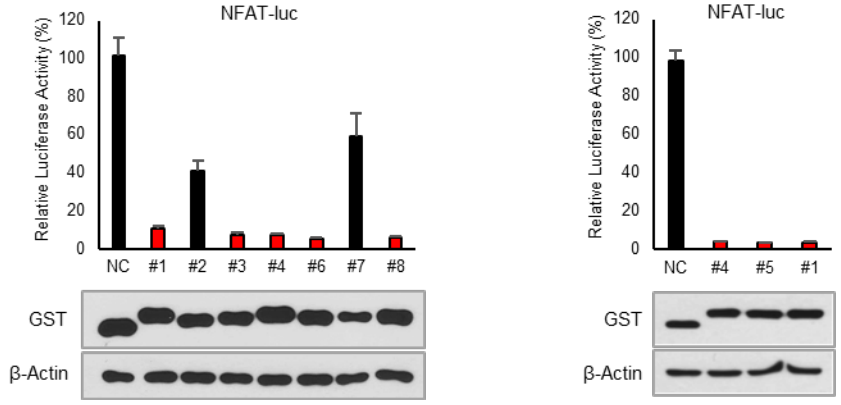

Supplementary Fig. 2 The role of the neighboring sequences around the "PxIxIT" motif a Sequence of CABIN1, VIVIT, and chimeric peptides. **b** Immunoprecipitation assays using Flag-CNAβ2 and GST-peptides in HEK293T. **c** Luciferase reporter assays were performed to measure NFAT transcriptional activity in Jurkat T cells expressing GST-peptides under PMA and Ionomycin treatment. All experiments were repeated three times and presented as the mean ± standard deviation. The expression levels of the peptides were confirmed by Western blotting. NC, negative control.

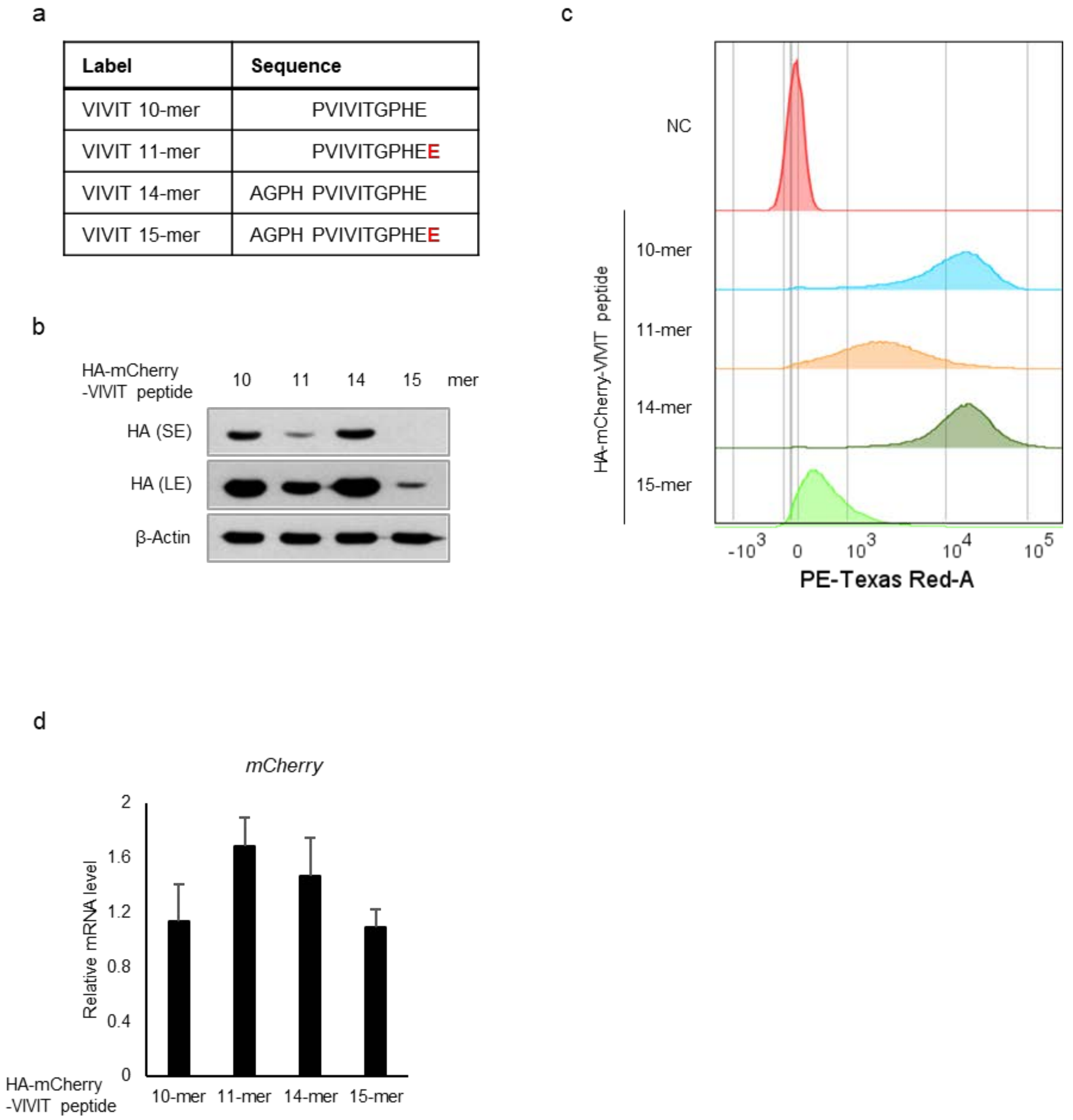

Supplementary Fig. 3 Protein stability of VIVIT peptides a Sequence of VIVIT peptides. **b** Protein expression levels of VIVIT peptides were detected with HA antibody in Jurkat T cells expressing HA-mCherry-VIVIT peptides. **c** mCherry expression intensity and distribution were analyzed by FACS in the Jurkat T cells expressing HA-mCherry-VIVIT peptides. ($5x10^4$ cells/sample) **d** mRNA expression level of *mCherry* was measured by qRT-PCR. (n=4) Values shown are the mean ± standard deviation. SE, short exposure, LE, long exposure, NC, negative control.

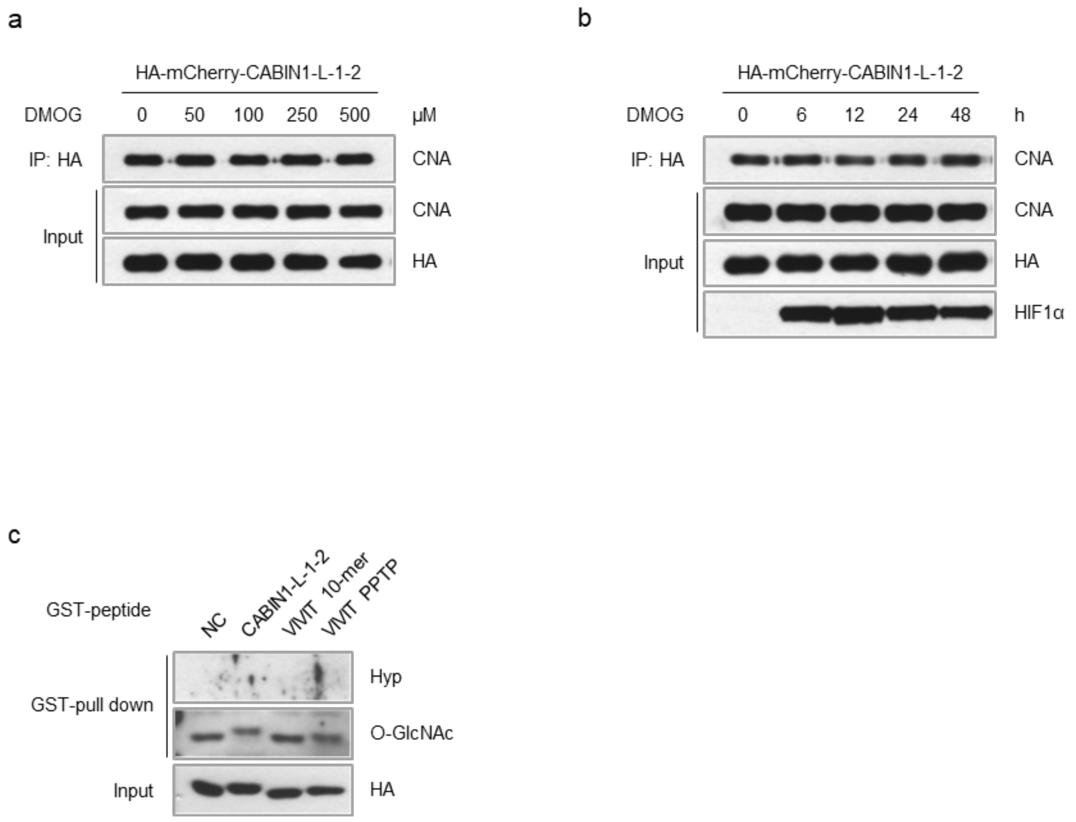

Supplementary Fig. 4 No hydroxylation or *O*-GlcNAcylation occurred in the "PPTP" sequence a Jurkat T cells expressing HA-mCherry-CABIN1 peptide were treated with DMOG in a dose-dependent manner for 12 h. **b** Jurkat T cells expressing HA-mCherry-CABIN1 peptide were treated with 500 μM DMOG in a time-dependent manner. Each cell lysate was immunoprecipitated with HA antibody and CABIN1 peptide-bound CNA was detected by Western blotting. HIF-1α was detected as a positive control for DMOG treatment. **c** HEK293T cells were transfected with GST-peptides and GST pull-down assay was conducted. Hydroxyproline and O-linked glycosylation of the peptides were detected by Western blotting. NC, negative control.

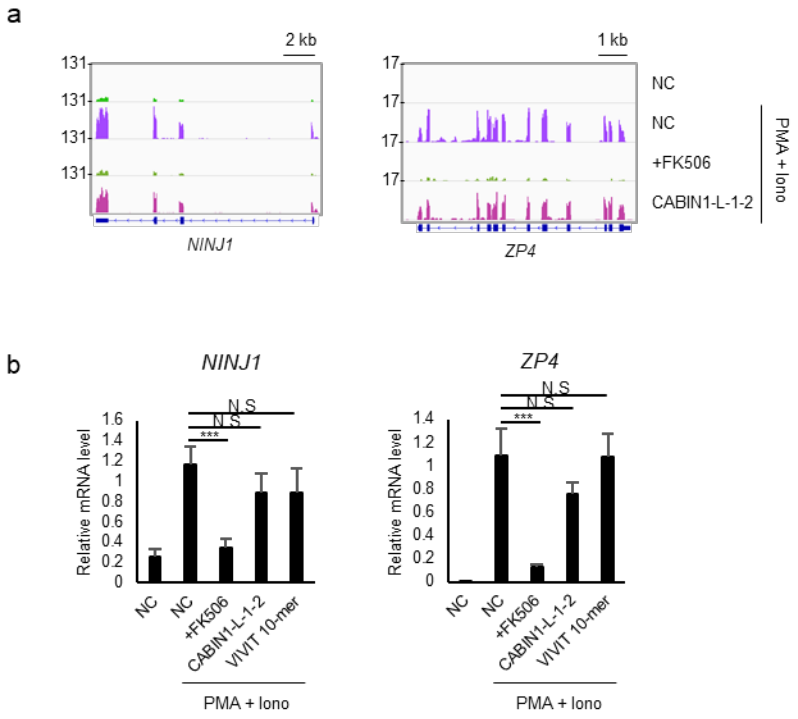

Supplementary Fig. 5 Putative calcineurin target genes that are not regulated via the calcineurin-NFAT pathway a mRNA expression levels of genes repressed by FK506 but unaffected by the CABIN1 peptide. **b** qRT-PCR data of genes of **a**. The experiment was independently repeated at least three times and presented as the mean ± standard deviation. Statistical differences were determined using one-way ANOVA. ***p < 0.001, NS, non-significant, NC, negative control.

2. Supplementary Tables

Supplementary Table 1 Primers used for real-time quantitative PCR

Gene	Sequence of Forward Primer	Sequence of Reverse Primer
IL2	ACAGCTACAACTGGAGCATTTA	TCAGTTCTGTGGCCTTCTTG
IL3	GCTGGACTTCAACAACCT	CAGACATGGCAGGAGATTT
CCL4	GTACGTGTATGACCTGGAAC	GAGATGTGTCTCATGGAGAAG
CCL20	GACTGCTGTCTTGGATACAC	TACTGAGGAGACGCACAA
CXCL8	GAACCATCTCACTGTGTGTAA	TGGAAAGGTTTGGAGTATGTC
NFATC1	CCGTTCACGTCAGTTTCTAC	GTTGGAGCAGGCTCATAATC
CD27	GCACTGTAACTCTGGTCTTC	GGGTTTGGAAGAGGATCAC
CD70	GATGGCATCTACATGGTACAC	GGTACAACCTTGGTGGAAG
NINJ1	CCTCATCTCCATCTCCCTT	GTTGACTACCACGATGATGAA
ZP4	GGAGACCGAGCAGTATATGA	CTGGACATTGATTGGGAGAG
mCherry	ACGGCGAGTTCATCTACA	TCAGCCTCTGCTTGATCT
18S rRNA	GCTTAATTTGACTCAACACGGGA	AGCTATCAATCTGTCAATCCTGTC
GAPDH	CCGTCTAGAAAAACCTGCC	GCCAAATTCGTTGTCATACC

Supplementary Table 2 Genes affected by PMA and ionomycin

NAME	NC/ FPKM	NC_P+I/ FPKM	FK506_P+I/ FPKM	CABIN1_P+I/ FPKM
PDGFA	1.497	26.809	3.25	8.169
BIN1	5.345	1.469	2.374	1.968
TAP2	1.126	5.044	5.187	6.702
TAL1	45.244	11.71	22.337	18.466
HIVEP3	11.136	18.008	20.041	20.545
ZNF827	0.975	3.401	1.521	1.372
SH2D1A	71.224	14.498	23.649	16.781
MIR6821	17.553	62.894	13.626	25.116
SLAMF7	0.276	10.24	3.121	3.544
CERKL	32.7	7.889	13.458	10.348
METRN	4.576	1.12	1.779	1.604
ZNF367	9.126	2.543	2.354	2.274
GKAP1	3.751	1.113	2.692	2.927
LOC100506688	4.117	1.142	1.625	1.704
NR4A3	0.024	4.559	1.927	1.977
PXYLP1	12.843	3.772	5.01	4.92
PLEC	5.129	16.749	21.747	25.453
SMOX	6.97	27.876	14.9	18.934
UBE2L6	30.923	127.161	164.565	167.347
EGR2	0.022	34.883	11.435	9
CAMK2D	1.999	13.185	5.855	6.138
APOBEC3F	1.328	6.423	5.861	7.066
ADORA2A	2.492	11.633	0.998	1.475
GTDC1	3.595	10.378	4.629	5.144
SPINT1	3.839	1.14	1.948	1.712
DUSP10	0.55	7.447	11.981	12.142
WARS	33.486	106.713	105.541	112.259
ITK	19.685	58.349	58.558	62.324
IQGAP2	9.764	1.968	4.857	3.477
ECE1	8.894	116.972	20.046	59.384
ADCYAP1	2.988	0.459	3.646	3.219
NFKB2	6.744	74.327	49.22	71.869
IRF2	5.852	18.15	34.99	34.829
TBC1D4	20.739	73.749	12.549	13.191
CSF1	0.848	13.43	1.706	2.594

BCL7A	60.161	14.095	12.185	12.277
LMNA	0.151	0.699	4.62	2.786
RCOR2	4.622	1.139	1.361	1.298
TCF3	72.022	25.169	39.165	36.607
PTPN6	22.163	77.092	20.906	20.806
FAM64A	14.202	3.009	7.456	6.008
CHPF	4.868	16.165	9.759	12.714
MAZ	235.37	81.339	112.102	99.474
SLC22A1	0.158	61.938	7.005	42.379
ZYX	8.696	29.119	127.794	85.489
ATPAF1	23.844	8.33	11.764	10.283
IL2	0	397.807	0.287	12.964
CCL3	0	52.239	0	0.502
NAA16	8.223	2.868	3.527	3.877
FOSL2	1.294	7.508	2.204	2.362
CXCL8	0.037	26.755	0.937	3.327
DBP	6.72	1.783	2.396	1.773
SERPINE2	1.408	5.558	3.936	3.911
PHLDB2	6.022	1.831	3.898	3.404
OSBPL5	11.263	1.794	3.354	3.123
CNN2	61.031	5.057	30.77	18.455
CDKN2D	29.191	8.631	17.642	16.801
PELI2	19.511	5.485	11.262	8.166
LUCAT1	2.321	7.089	12.516	9.183
CBS	11.413	3.102	3.935	3.916
ABTB2	0.787	5.914	2.549	6.382
TAPBPL	9.485	25.582	19.59	20.693
CYTH1	56.655	19.659	33.17	30.15
XCL1	0	49.113	0	1.144
PAM	1.154	5.577	1.483	1.934
GLRB	7.592	2.451	1.62	1.354
KLF12	6.701	2.025	6.506	4.377
PKM	443.853	2211.937	1736.037	1838.428
MAMLD1	0.598	6.963	5.995	9.949
VAV1	34.441	11.911	30.218	22.182
APOBEC3G	0.737	22.838	8.803	9.231
CFLAR	1.799	3.856	3.438	3.022
ZNF521	4.277	0.956	2.011	1.965

PRNP	13.132	43.872	39.685	41.922
PPIF	58.328	188.301	50.291	106.533
CHPF2	12.181	33.063	36.42	34.939
BEND6	3.986	1.153	1.416	1.409
MAST4	0.876	5.954	3.498	5.841
SORBS1	0.196	5.08	0.4	0.511
IL21R	0.1	34.712	2.244	5.255
STAT3	17.67	52.79	83.385	74.439
LTBP4	7.304	33.466	8.427	9.568
PSMB10	31.284	85.264	104.49	125.268
VAT1	100.917	34.64	44.696	40.336
LOC653712	6.512	1.854	4.163	2.808
IQCJ-SCHIP1	1.402	4.253	1.657	1.234
NFATC1	4.511	15.287	3.793	3.916
EHBP1L1	7.342	2.399	7.073	5.023
CENPM	44.679	13.062	13.201	14.415
ASNS	10.082	29.993	20.436	19.026
LOC101927497	24.857	7.313	9.413	10.444
SSBP2	28.793	4.463	7.127	5.549
MAP3K7CL	0.28	3.831	2.247	2.03
LPAR6	0.354	1.601	8.449	4.344
TRIB1	0.189	16.309	5.422	4.108
HPGD	8.191	1.884	6.064	3.392
PPP1R16B	2.67	37.059	29.659	26.306
PLS3	0.019	4.717	0.052	0.37
LRRC20	12.151	2.17	3.843	2.839
NEDD9	1.089	4.206	3.748	4.972
IL15	0.235	1.236	2.654	3.16
RHOU	3.829	1.044	1.51	1.378
RAB37	3.573	0.196	2.455	1.369
ACAP1	41.657	13.82	35.261	26.535
B2M	411.858	1193.352	1155.981	1302.512
MYB	89.343	25.529	58.524	48.107
VAMP1	9.566	50.97	14.793	14.752
ADAMTS17	5.733	1.475	0.843	0.882
THEM6	11.312	3.511	3.232	2.542
RCBTB2	10.697	1.899	13.008	7.77
PKIG	21.186	5.694	12.411	11.17

HCAR1	0	11.451	7.167	7.943
CEP70	34.21	9.737	14.887	12.745
SLC35E4	0.887	9.069	0.952	1.517
CD82	26.521	419.996	171.933	222.109
LEF1	186.267	37.079	74.102	48.109
SYK	15.907	4.047	7.044	6.222
ALDH1A2	42.14	4.551	7.041	6.66
ITGA4	88.754	23.254	39.099	31.73
SH3TC1	11.764	0.939	1.562	1.895
CD274	0.244	24.463	7.13	15.92
ARPP21	5.805	0.431	1.29	0.7
OPTN	1.609	7.963	12.958	14.333
CPD	8.281	35.158	23.208	26.602
LDLRAD4	17.909	3.055	6.21	4.769
TNFSF14	0.045	69.958	1.023	4.255
KIR3DX1	0.01	0.394	3.048	3.992
PDIA6	53.625	142.05	123.669	128.39
HLA-A	48.846	144.622	181.069	226.558
TIAM2	0.255	4.095	0.27	0.301
GSE1	39.875	14.157	17.37	14.556
FOSL1	0.132	12.971	10.499	12.705
UHRF1	34.265	7.455	8.288	6.903
TMCC3	0.053	0.478	4.527	7.278
ZNFX1	8.122	24.811	16.708	19.096
FXYD5	25.643	101.352	41.346	42.856
DUSP6	0.552	5.792	9.728	12.914
IL4R	2.023	39.885	9.842	12.536
ACSF3	33.65	6.108	22.755	15.621
CD200	0	3.759	0	0.094
KIAA0247	4.869	13.963	8.032	8.83
ANTXR2	1.504	20.776	53.159	55.18
POU2AF1	1.84	36.298	2.274	2.971
IKZF2	24.637	6.145	8.561	8.132
IRF1	13.332	118.876	141.607	152.425
GPR17	3.126	0.144	0.258	0.181
TNFRSF10B	7.441	37.653	25.474	36.431
N4BP3	0.544	5.501	1.739	2.499
MCM2	95.73	28.688	22.07	23.634

SASH3	86.466	26.7	59.349	46.514
GPC2	7.54	2.341	5.785	4.85
NTRK1	0.167	7.941	0.836	1.657
THEMIS	35.003	7.905	33.406	35.627
E2F7	11.171	2.941	4.531	4.018
STMN3	24.035	5.042	10.423	7.407
LGMN	4.67	1.311	1.42	1.976
MAFF	1.24	13.462	16.977	16.464
CBX2	7.091	1.05	2.786	2.454
LINC01260	17.932	2.402	7.672	7.215
LIMS2	24.38	4.029	4.122	4.304
KLF10	3.03	16.287	25.627	15.364
TIAM1	4.518	14.204	8.466	9.551
ETS1	90.462	23.146	50.032	38.627
MGAT4A	17.02	4.173	8.906	8.882
SPRY4	0.076	7.939	9.613	7.991
GLRX	33.473	8.559	25.315	21.013
MAL	11.053	0.86	2.664	2.026
RASGRP2	16.42	1.08	3.002	2.335
FBLN2	13.535	3.433	3.348	3.01
SLC29A4	5.261	1.576	1.68	1.658
TCF7	82.711	10.7	13.065	11.184
ARHGAP4	32.153	10.672	15.97	16.216
SCHIP1	1.25	3.933	1.535	1.333
IFIT3	0.099	2.372	6.495	6.682
CD1E	45.421	3.154	4.975	3.521
NSMF	1.169	4.89	3.164	3.265
QPRT	26.609	7.071	13.402	10.818
FAT1	29.711	10.233	13.036	11.964
CCNE2	19.544	4.642	6.298	5.206
NCKAP1	3.582	11.313	8.582	10.157
ST3GAL6	1.195	3.863	4.018	5.239
CDKN1A	0.998	42.067	39.264	62.513
LAIR1	12.869	1.724	8.298	7.19
GXYLT2	13.458	2.813	5.443	4.24
RNF212	3.075	1.394	1.059	1.401
AVPR2	3.072	0.571	1.21	1.492
MXD3	22.044	21.527	17.861	20.08

CREB3L2	1.952	6.725	5.62	5.857
PRDM8	1.421	12.326	39.913	40.783
DSTYK	2.356	7.198	3.501	3.595
JAK2	2.788	26.566	5.248	8.45
PDCD1	0.327	4.738	1.377	1.974
ADA	405.953	51.958	74.531	73.254
ZP4	0	6.38	0.462	5.182
LOC101926963	1.636	7.12	4.541	4.742
FYN	14.671	58.084	16.523	15.512
SEPT6	90.554	29.289	55.091	44.841
CCND3	76.176	19.271	30.402	25.396
TAGAP	3.351	128.498	4.352	8.731
NT5E	0.462	15.919	8.91	12.217
GFOD1	2.283	12.861	4.187	6.312
RASGRP1	27.453	7.034	4.464	3.909
SEPT3	3.991	0.884	1.089	0.994
PEX5L	5.583	1.531	1.428	1.299
GZMB	0.476	3204.801	513.528	909.046
PLAC8	6.697	1.514	3.158	1.896
ZNF480	15.131	3.391	7.174	6.823
MSMO1	17.034	46.91	59.655	52.664
ANKEF1	3.896	0.702	3.081	2.575
FADS2	108.804	34.879	67.903	48.612
NFKB1	16.458	71.749	34.242	44.661
LIF	0.063	3.697	0.143	0.231
ASB13	10.32	2.756	2.277	2.543
SOX4	95.745	20.819	76.17	55.775
TNFAIP3	4.333	55.781	25.168	35.424
EFHD2	34.309	137.802	180.233	234.38
NDRG2	5.775	0.944	2.881	2.149
COL27A1	4.149	17.216	4.463	5.729
IL27RA	2.341	9.341	10.817	10.764
ZFP36L1	5.72	143.376	153.648	136.279
PHACTR2	1.659	9.467	2.927	2.791
POU2F2	0.87	12.177	3.317	4.109
EMP1	0.156	8.12	4.613	4.589
ATP6V0A4	0.042	2.687	21.385	18.558
POTEE	3.342	1.022	1.42	1.196

APH1B	5.907	1.729	5.12	4.206
SPRY1	2.079	12.391	4.074	4.901
MT2A	71.941	413.047	369.658	409.501
CARHSP1	39.677	11.699	20.082	14.562
AFF2	7.635	1.272	4.374	3.175
SERINC5	13.434	3.2	3.184	3.312
PDGFC	17.949	2.362	2.292	2.913
BCL2L1	31.375	113.956	39.402	41.852
STX1A	1.467	10.488	16.1	13.882
SBK1	9.277	2.256	9.239	5.945
IL10RA	0.471	1.592	2.828	3.208
SERPINB8	8.633	26.053	23.788	23.508
IL15RA	0.203	3.751	5.979	7.481
TIAF1	15.536	5.204	12.479	9.375
SEPT9	97.462	23.985	55.265	39.741
PTPN7	43.729	160.438	93.59	78.61
XCL2	0.056	70.747	0	2.074
MIR3652	96.162	486.958	547.182	567.901
KREMEN1	6.452	2.248	3.122	2.958
NDRG1	5.346	28.817	126.361	128.386
MICAL2	0.865	3.866	1.763	1.84
TBKBP1	2.812	38.348	16.894	22.205
KCNK1	10.828	36.893	10.967	13.139
SERTAD1	3.825	17.874	16.162	14.426
SH2D2A	2.277	41.819	20.658	22.586
ODC1	133.175	391.769	245.466	374.309
SIVA1	172.026	51.28	50.269	51.964
BAHCC1	9.923	0.482	1	0.873
APOL1	0.065	1.03	4.036	4.498
FKBP5	24.757	8.891	8.687	7.791
DCBLD2	2.644	12.227	13.786	15.588
FLI1	19.369	6.572	13.241	10.752
DNAJB5	3.056	16.902	19.238	19.35
CREB5	0.031	4.533	0.577	1.966
APOBEC3B-AS1	0.045	16.621	13.251	20.386
SLAMF1	0.042	5.663	0.217	0.275
C11orf74	10.295	0.715	0.365	0.392

SMAD1	7.505	2.417	4.873	3.995
NSG1	11.29	2.308	4.302	3.929
DTX1	18.177	2.218	4.414	3.245
GFRA1	3.744	0.902	0.991	0.76
CECR1	6.961	1.828	5.304	3.984
ALOX5AP	22.944	7.373	92.971	39.735
XRCC6BP1	20.482	6.203	9.744	9.204
NOTCH3	6.853	0.53	0.902	0.859
RUNX3	4.704	25.871	23.624	28.166
LEF1-AS1	2.965	1.506	3.747	2.388
MIR6775	12.409	50.527	19.643	21.57
RNF19A	6.138	25.66	12.334	12.101
C10orf55	0.1	11.26	7.632	15.779
STAT2	7.544	21.144	37.805	36.117
ANO8	4.062	0.957	3.26	2.721
STAU2	11.237	4.171	7.124	5.719
NME4	42.216	14.381	16.395	17.501
PCNA-AS1	206.038	72.084	70.937	71.806
BCL11B	17.763	3.329	3.688	3.353
ATP2A3	113.023	15.616	47.81	36.996
ARHGEF3	6.146	31.337	8.637	8.674
CHST12	23.741	8.104	13.527	12.639
NLRC3	7.329	2.409	7.789	5.507
SPSB1	0.944	4.094	10.137	9.593
RRAS2	5.694	15.372	18.257	20.023
LRRC8C	2.556	31.63	10.058	11.753
RUNX1	73.877	25.016	37.963	28.867
YPEL1	7.804	2.488	5.628	4.38
SMPD1	1.941	7.046	20.599	18.206
CSF2	0	23.497	0	0.314
TPM4	175.429	60.961	202.311	178.336
ITGAL	17.143	2.42	10.212	7.674
SLC35G2	3.937	12.866	10.118	11.385
ATHL1	11.436	2.861	4.597	6.014
BCL6	1.073	9.143	15.181	15.55
DTL	26.496	5.74	5.862	5.275
SGK1	0.854	22.157	76.683	105.258
TPST2	10.644	42.955	21.077	29.147

HLA-B	14.546	137.409	116.079	160.359
XIRP1	0	63.877	6.348	13.281
SRGN	4.303	14.597	10.812	9.743
NREP	33.624	8.235	9.925	6.879
CHEK2	23.817	6.012	8.907	7.751
TCF12	80.871	23.735	34.798	29.903
GNG4	2.073	10.63	5.365	7.415
SLC27A2	4.292	13.006	41.996	41.908
AGK	14.395	39.056	14.029	14.306
GAD1	0.317	3.943	2.246	2.238
NRP1	0.177	3.835	14.572	14.016
TRIM21	9.766	26.937	45.856	42.08
KIAA0101	43.447	11.336	21.727	16.766
CD97	10.97	49.481	19.394	21.496
TLE1	5.785	1.657	1.864	1.647
PPP1R15A	10.908	54.597	77.478	76.265
IRF4	0.034	8.439	0.423	2.092
RTN4R	5.402	0.969	2.449	1.548
NPDC1	3.008	8.759	4.328	5.64
ZNF280D	5.448	1.494	2.345	2.132
IRF7	4.722	14.286	21.167	21.372
CBFA2T3	27.65	5.839	12.236	11.821
MAGEA4	1.603	5.263	3.908	3.775
CCL20	0	59.393	0	1.279
TNFSF10	1.024	9.324	24.883	49.452
EGR1	2.036	64.209	218.444	123.294
CABP1	1.702	5.834	1.201	1.689
RORA	4.213	1.245	2.502	1.998
BCL2A1	0.067	222.759	21.118	51.928
NCOA3	3.871	14.211	9.949	11.775
MFAP4	3.474	0.577	0.663	0.663
LMO1	4.098	1.424	1.094	0.954
TGFBR2	6.333	43.071	36.615	36.254
SCG2	1.46	54.619	159.394	86.711
MZB1	101.866	23.221	22.015	23.597
TTLL1	7.109	2.235	3.69	3.295
CHRNA3	6.949	2.269	4.293	3.499
ZNF704	0.107	4.985	0.014	0.183

MCTP2	0.583	3.508	5.326	3.187
BCL3	0.067	1.132	14.57	17.15
CD70	1.101	61.146	3.005	7.606
ARHGAP25	2.976	0.812	3.447	2.157
RB1	71.078	24.713	62.321	49.538
PI16	24.532	2.217	17.07	9.625
TDRD9	1.428	25.902	1.245	1.888
CCDC15	6.226	2.079	3.535	2.926
PDLIM1	21.843	3.522	6.874	5.135
ZFP36L2	30.948	8.275	13.343	9.774
CD1B	25.556	4.688	4.115	4.733
ARHGAP31	1.341	8.14	10.889	11.177
TBC1D5	9.718	1.789	4.088	3.021
BIRC3	2.957	70.496	30.767	58.303
CBLB	3.959	11.37	8.881	7.757
IL21R-AS1	0.048	23.616	1.927	3.991
LINC00659	0.604	3.527	10.239	9.828
TRAF3IP3	25.055	4.597	15.012	11.237
SLAMF8	0.199	3.509	5.282	5.828
WDR76	23.051	8.198	8.368	8.215
GPR68	0.558	2.704	8.916	11.224
CXCR4	188.803	26.517	38.979	34.014
MIR22HG	1.343	6.088	9.248	8.63
DENND5A	8.353	30.108	24.792	37.896
ATP6V1B2	33.172	129.395	50.672	60.661
ZNF652	3.976	1.292	2.631	2.469
SPTBN2	4.048	0.792	2.135	2.053
RGS2	0.977	9.667	3.365	3.39
NOTCH2	3.645	11.923	10.308	12.916
CXCL11	0	0.648	0.695	3.793
C21orf58	12.045	3.947	5.847	6.073
CCL3L3	0	6.524	0	0.079
GDPD5	4.329	12.625	29.626	39.139
VANGL1	21.457	5.713	9.553	8.842
TM4SF1	0	3.268	0.439	0.907
PARP9	6.919	50.016	84.306	84.386
CTTN	0.428	16.075	11.037	15.629
VGF	0.282	11.869	59.749	54.294

CCDC71L	2.517	15.309	13.763	16.319
RGS16	0.077	29.102	1.172	5.595
LINC00426	3.591	0.365	1.041	0.675
IL3	0	229.054	0.062	6.298
CFD	3.255	0.818	0.937	1.22
PXMP2	18.061	5.569	5.624	7.334
LAX1	8.344	24	8.052	7.578
MCM4	89.017	30.965	26.368	27.291
SLC25A45	7.535	1.819	3.26	2.626
DOK2	8.332	36.459	27.372	27.542
JUNB	12.877	53.734	55.036	56.126
MYO7B	59.265	8.343	15.796	12.661
DUSP2	18.632	472.111	123.613	97.06
IGLL1	34.29	2.82	3.429	3.244
C1orf233	4.195	0.525	0.79	0.674
TBC1D10C	17.466	4.364	23.871	15.764
CYB5RL	3.61	1.139	1.807	1.449
DPYSL2	15.045	5.427	11	9.177
GLIPR2	16.149	5.189	15.84	11.773
RGS3	0.249	5.068	2.142	2.303
BHLHE40	2.499	6.901	6.563	9.368
PPFIBP1	4.095	10.885	12.205	12.117
GNAQ	43.005	11.031	21.057	17.078
XYLB	8.978	2.856	1.506	2.311
EPAS1	0.188	0.797	5.846	4.197
NKX3-1	23.419	6.852	7.464	8.412
SERPINB2	0.28	85.882	2.905	14.103
ADD3	21.964	4.318	13.6	9.536
SH2B3	7.761	23.644	13.899	14.803
CCDC64	5.477	33.545	10.044	11.039
C9orf9	12.861	3.075	7.304	6.009
FST	0	15.319	2.244	6.872
RORB	3.524	0.72	3.782	3.292
OSBPL3	8.455	22.99	24.517	23.298
XBP1	17.617	54.64	43.828	48.525
SLC2A3	2.444	19.284	45.525	40.147
ARHGEF6	55.593	12.62	35.366	27.254
LRRC8B	2.89	28.699	3.555	4.298

ENHO	3.463	0.34	1.746	1.223
KIF3B	7.124	25.597	13.87	14.583
STAT5A	1.417	9.105	3.347	3.055
CD1A	39.249	3.977	4.589	4.51
TNRC6C	10.066	3.479	4.711	4.485
AQP3	36.184	11.327	66.037	52.98
SHC4	0.069	4.391	0.663	0.686
TMTC4	7.082	1.794	2.677	3.005
DNPEP	17.801	50.94	31.154	43.482
MRPL39	38.485	238.099	132.452	188.834
PLAU	0.66	61.729	45.171	82.517
NEXN	0.226	2.551	3.661	5.587
IL4I1	5.799	6.34	7.078	7.279
HAPLN3	1.442	48.075	48.4	58.345
RAP1GAP	6.799	2.193	1.878	1.799
CXCL3	0.245	4.241	3.147	7.007
TUBB3	11.952	2.205	9.713	5.423
C16orf46	1.017	3.001	2.655	3.705
PMEPA1	34.688	2.175	3.548	2.393
TSHR	7.258	0.256	1.535	1.574
FAM46C	6.988	2.032	5.191	5.693
CD1D	17.887	3.263	2.553	3.093
PTK2	5.74	20.624	13.215	13.391
ASAP1	4.678	18.37	7.765	9.052
PTPN22	8.928	45.38	6.37	5.119
IER2	19.131	59.903	181.539	126.884
CD83	14.024	46.169	35.907	41.173
TNFRSF4	2.597	31.683	2.933	11.916
NRROS	57.345	20.402	36.775	26.059
LYPD3	1.545	4.832	2.727	2.662
OAS3	4.289	11.893	13.085	18.24
LRRC45	14.257	3.147	4.142	4.264
DHFR	20.499	7.117	7.656	6.668
NDFIP1	19.638	54.615	22.485	24.432
IFFO2	1.647	5.162	2.752	3.163
IL16	1.427	6.75	10.552	8.304
TRAF1	0.528	46.848	11.176	24.962
HSP90B1	148.345	846.287	877.39	910.415

CNR2	4.165	0.223	0.674	0.552
SIX6	52.35	13.291	15.688	19.444
IMPA2	11.686	3.007	3.015	2.882
IGSF9B	2.045	8.871	7.175	8.992
PWWP2B	4.987	1.248	2.769	3.446
GPR108	17.207	47.945	31.682	37.394
CORO2A	5.418	1.42	1.862	1.623
XAF1	1.876	8.299	15.924	16.914
NEIL3	26.1	5.801	9.418	8.336
CXCL9	0	1.581	0.839	7.221
NR4A1	2.716	25.797	52.553	34.85
LAMP3	8.064	31.61	6.412	9.828
GAMT	27.381	9.875	10.915	11.98
TRIM22	0.102	0.842	2.908	3.316
TSPAN2	4.997	0.893	2.414	2.119
APOL2	11.71	63.794	89.267	102.003
LOC729603	1.468	5.071	2.179	3.329
HDC	0.024	14.173	26.3	23.209
SECISBP2L	6.651	19.048	13.57	12.883
PKIA	6.444	49.019	13.962	18.138
CD55	3.587	39.451	6.015	12.082
PHEX	0.188	17.377	0.213	0.786
GRK5	5.451	21.464	8.481	8.975
PLEKHO1	55.893	18.221	47.519	36.376
FMNL2	2.395	6.813	2.726	3.019
TTC28	4.28	1.23	2.798	1.926
CCL4L2	0	12.758	0.087	0.328
CXCL10	0	1.106	0.352	6.288
CSRNP1	2.387	26.898	26.052	23.093
PHF7	6.667	2.026	3.968	3.075
ATXN1	0.733	3.381	1.798	2.083
CHRNA9	4.187	1.099	4.046	2.572
LINC00528	6.661	1.762	5.759	5.927
GRAP2	30.381	10.47	19.094	11.197
CRIM1	0.268	3.029	0.305	0.333
FAM111B	13.399	3.849	5.291	4.22
LAIR2	0	1.933	2.203	3.695
GZMH	0.283	45.057	7.694	13.893

GNG2	26.471	8.896	25.67	17.754
ARHGAP23	4.013	1.003	2.576	1.985
TNFRSF14	1.345	4.446	12.35	12.09
E2F2	13.902	2.827	3.317	3.419
SPRY2	4.541	18.913	16.882	17.876
LDLRAP1	3.217	0.374	0.778	0.878
SEMA7A	3.218	42.26	26.567	34.623
HHIP	18.16	3.865	9.345	6.793
FASLG	0.034	7.22	1.518	1.715
HIVEP2	2.983	22.441	7.494	9.184
SYTL1	14.703	4.911	15.004	12.555
SH3RF2	0.546	8.116	27.941	32.15
PODXL	4.505	0.916	1.86	1.509
SREBF1	27.932	7.792	14.076	12.747
DFNA5	2.024	13.031	12.016	9.395
TIMP2	9.09	1.3	1.948	1.952
MIR6891	3.784	30.185	24.428	31.815
KIAA0922	40.863	14.062	12.92	12.911
GEM	0.129	7.322	2.495	2.963
AGO2	7.51	21.395	13.242	14.51
SH2D5	0.508	1.613	1.469	3.557
APOBEC3B	0.164	46.046	41.16	59.895
ISM1	0.798	56.893	0.726	3.737
UHRF1BP1L	4.289	13.987	7.474	7.889
SOCS1	0.361	5.77	9.708	11.547
ADAM19	0.115	3.123	5.532	4.257
ERO1LB	1.452	4.009	5.101	4.345
ICAM1	1.13	8.163	12.704	12.914
ADORA2B	2.309	10.507	8.219	6.969
SOCS3	0.412	6.909	8.922	9.392
KIF1B	3.377	17.954	11.727	13.254
FBXO34	11.735	48.015	14.612	18.386
IRF9	4.818	29.184	39.109	39.375
SOSTDC1	11.831	1.679	8.472	7.34
IFNG	0	28.865	3.8	8.858
LINC00152	3.473	60.002	32.822	31.801
E2F1	30.985	10.056	7.783	8.062
NDFIP2	3.082	14.277	13.13	12.598

MAP2K3	21.676	144.057	41.815	43.237
KCTD11	4.27	1.086	3.399	2.303
USP30-AS1	0.48	3.084	6.464	9.017
ARC	0.021	22.76	9.966	10.186
FYB	22.142	5.913	11.107	10.258
PRKCA	40.602	9.875	14.104	11.132
APOBEC3C	3.797	16.567	16.851	14.323
BACH2	2.387	57.828	29.7	36.118
TRAT1	13.116	1.838	7.968	5.507
RMI1	18.909	6.379	8.984	8.198
GBP3	2.313	34.082	69.945	68.757
TBC1D20	11.844	35.808	17.7	18.427
HHIP-AS1	66.764	14.584	32.468	29.854
CDCA7	92.001	24.768	26.911	21.851
PDE3B	14.442	4.977	9.108	5.194
ARAP2	2.197	10.711	15.321	13.458
C1QL1	3.175	0.772	1.444	0.756
CCL1	0	9.86	0	0.415
PIM3	37.434	180.382	42.827	72.895
CBX6	19.627	62.434	42.006	65.604
RAG2	10.016	0.244	0.173	0.385
RDH10	4.125	19.889	5.856	5.524
SLC2A4RG	5.881	1.461	2.367	2.168
GBP5	0.062	130.751	197.514	262.945
ST8SIA4	3.394	9.53	11.077	8.921
MBOAT2	5.378	1.806	3.773	3.202
CHI3L2	160.778	54.252	58.214	51.627
SERPINE1	0	2.076	6.785	4.431
SIT1	51.291	7.298	48.56	34.287
GPR183	0.281	1.519	5.776	4.735
PHLDA1	0.64	30.9	65.703	52.893
GPR3	1.185	10.707	7.96	9.964
MMP10	0	188.369	11.973	22.372
SLC7A5	104.518	500.415	207.842	226.44
MYO1G	64.829	11.602	98.59	65.452
APOL6	2.603	27.312	46.937	46.762
FURIN	7.171	19.805	23.264	29.008
CRTAM	0.104	100.175	3.325	9.51

PARP14	7.365	40.426	65.432	72.911
ID2	2.484	27.855	13.425	15.335
ENC1	2.218	14.108	9.299	9.268
NFKBID	3.606	10.726	6.668	8.294
PELI1	5.755	16.085	8.175	8.465
LINC00539	11.913	1.403	5.004	2.351
NFKBIA	9.923	79.3	39.193	70.837
CCND2	0.321	6.436	0.558	0.99
IL2RA	0.01	16.919	5.833	10.904
SH3BGRL3	111.636	355.335	408.163	351.357
SELPLG	21.352	1.378	5.062	3.724
CXCR3	0.604	14.322	0.503	1.803
NDST3	10.786	1.405	10.369	5.621
PIDD1	11.655	3.683	5.28	4.471
GINS2	56.457	18.741	16.935	16.516
IRF8	1.168	14.813	1.19	1.914
NFE2L3	8.648	31.259	15.944	28.495
HSP90B2P	2.295	9.218	8.45	9.619
IFIH1	2.155	8.429	11.544	12.37
REL	2.654	20.219	4.749	5.699
SAMD1	30.407	9.053	15.561	12.809
EVI2A	1.069	11.844	15.078	10.047
TBX21	0.171	57.479	24.784	39.746
PCBP3	0.923	3.111	4.527	4.642
CCL4	0	247.287	0	4.062
SMIM14	4.316	1.197	7.677	3.572
KCNK5	3.84	14.865	3.906	4.904
ST3GAL6-AS1	1.309	9.671	9.039	8.479
TMEM229B	1.854	7.079	6.813	7.747
ACTG1	1354.665	452.78	1002.221	799.981
JAK3	1.607	6.336	8.03	8.871
THY1	11.123	2.912	3.994	3.297
ATP2B4	18.152	1.829	16.449	10.46
STAT1	15.878	317.544	611.139	527.58
GBP1	0.504	27.863	70.024	87.487
RELB	1.091	50.179	29.337	40.689
PTPRE	5.085	14.58	14.031	14.28
GBP4	0	2.965	4.384	9.209

MAP2K6	6.52	1.459	1.149	0.955
CD69	2.921	416.388	125.597	121.846
PMAIP1	10.951	37.079	26.095	37.469
CD1C	8.708	1.918	3.288	2.59
TSPAN7	145.387	35.603	44.718	42.604
ENPP1	0.211	9.218	124.248	51.27
GBP2	0.015	45.346	27.591	38.881
METRNL	0.141	6.604	7.501	7.881
NXN	12.588	1.19	2.574	1.571
DUSP5	0.991	95.69	116.404	157.077
PLK3	4.432	14.807	5.257	7.036
CTDSPL	3.213	1.033	1.758	1.836
NPY	0	4.94	4.771	4.799
TNFRSF12A	0.792	3.349	8.559	6.523
DNAJC1	5.462	19.836	13.765	13.844
TM7SF3	62.693	19.435	29.483	25.052
TNS3	0.008	13.196	0.678	1.418
STX11	0.534	5.108	2.733	2.972
TUBB2A	4.011	18.285	7.594	10.912
VASH2	8.763	2.383	5.761	4.245
RAG1	46.66	0.231	0.251	0.265
S1PR1	5.222	1.058	0.737	0.461
CD27-AS1	3.088	14.958	5.11	4.613
SLIT1	17.333	2.481	2.6	2.815
C3	0.006	3.907	0.489	1.329
DPY19L2P2	6.664	2.117	2.937	2.643
CCL4L1	0	13.27	0.087	0.328
BAI2	4.519	0.893	1.395	1.079
AIM1	26.38	7.777	37.047	27.486
NFKBIE	4.506	15.504	11.399	11.793
HSPA5	78.621	501.993	373.499	449.173
NKD2	15.011	3.784	3.385	3.105
SLCO3A1	5.528	1.725	3.481	3.095
IER3	0.506	91.488	31.73	26.899
CLEC2B	5.555	54.539	60.911	54.119
NINJ1	10.319	76.622	11.769	54.452
EIF2AK3	5.111	15.035	9.332	9.816
ZC3H12A	3.335	23.815	13.36	17.547

MIR4435-1HG	4.149	31.674	17.138	16.303
CCL3L1	0	6.448	0	0.079
ABCB10	51.637	9.932	10.164	9.751
NT5M	7.67	2.289	1.582	1.643
MCM5	104.109	31.862	37.057	33.895
DTX3L	8.459	36.101	48.022	48.261
DNAJB11	40.794	111.886	107.644	109.967
LINC00977	10.816	0.925	0.741	0.266
APOBEC3D	2.393	13.069	10.187	8.981
RAP1GAP2	3.998	0.092	0.368	0.285
PPM1H	11.904	2.973	4.492	4.178
TNFSF9	1.393	7.107	2.568	2.651
PEAK1	3.018	12.974	5.442	9.633
BATF3	18.188	49.098	36.514	45.085
POU3F2	3.711	0.566	1.044	0.696
CST7	3.315	14.15	2.338	3.175
MIR221	2.007	21.479	10.429	10.092
CASC15	5.894	1.102	4.027	2.572
CDT1	39.057	12.4	11.013	11.831
FOXO1	1.167	8.247	1.77	3.736
IL22	0	1.6	3.116	3.136
BTG1	4.391	33.793	22.099	23.454
ATG14	5.727	30.239	7.863	11.252
NR4A2	0	10.173	0.04	0.356
NAMPT	8.894	108.843	34.628	54.272
FOS	0.145	3.108	7.329	4.53
C10orf54	0.84	7.506	0.382	0.487
YBX2	3.976	1.141	1.555	1.594
LIME1	42.247	6.433	15.749	11.879
BAIAP2-AS1	3.939	0.977	2.72	2.144
CBX4	24.877	7.596	12.478	11.578
IL23A	2.262	20.398	12.207	17.874
EPHA3	7.734	2.853	2.019	2.536
MYO1D	4.114	11.118	4.59	5.995
JUN	1.525	5.925	3.835	3.58
APOBEC3A_B	0.029	11.374	10.242	14.665
STARD4	8.464	23.37	20.118	16.105
NLRC5	2.969	26.889	38.784	37.7

ETV5	0.596	9.791	15.576	13.264
KBTBD11	6.611	2.011	2.434	2.772
CD27	2.525	27.462	3.098	3.4
KSR1	1.826	8.146	4.79	5.998
KLF2	5.551	0.296	1.971	1.375

Supplementary Table 3 Genes in Cluster III

NAME	NC/ FPKM	NC_P+I/ FPKM	FK506_P+I/ FPKM	CABIN1_P+I/ FPKM
PDGFA	1.497	26.809	3.25	8.169
ZNF827	0.975	3.401	1.521	1.372
MIR6821	17.553	62.894	13.626	25.116
ADORA2A	2.492	11.633	0.998	1.475
GTDC1	3.595	10.378	4.629	5.144
TBC1D4	20.739	73.749	12.549	13.191
CSF1	0.848	13.43	1.706	2.594
PTPN6	22.163	77.092	20.906	20.806
IL2	0	397.807	0.287	12.964
CCL3	0	52.239	0	0.502
FOSL2	1.294	7.508	2.204	2.362
CXCL8	0.037	26.755	0.937	3.327
XCL1	0	49.113	0	1.144
PAM	1.154	5.577	1.483	1.934
PPIF	58.328	188.301	50.291	106.533
SORBS1	0.196	5.08	0.4	0.511
IL21R	0.1	34.712	2.244	5.255
LTBP4	7.304	33.466	8.427	9.568
IQCJ-SCHIP1	1.402	4.253	1.657	1.234
NFATC1	4.511	15.287	3.793	3.916
PLS3	0.019	4.717	0.052	0.37
VAMP1	9.566	50.97	14.793	14.752
SLC35E4	0.887	9.069	0.952	1.517
TNFSF14	0.045	69.958	1.023	4.255
TIAM2	0.255	4.095	0.27	0.301
FXYD5	25.643	101.352	41.346	42.856
CD200	0	3.759	0	0.094
POU2AF1	1.84	36.298	2.274	2.971
NTRK1	0.167	7.941	0.836	1.657
SCHIP1	1.25	3.933	1.535	1.333
DSTYK	2.356	7.198	3.501	3.595
JAK2	2.788	26.566	5.248	8.45
FYN	14.671	58.084	16.523	15.512
TAGAP	3.351	128.498	4.352	8.731
GFOD1	2.283	12.861	4.187	6.312

LIF	0.063	3.697	0.143	0.231
COL27A1	4.149	17.216	4.463	5.729
PHACTR2	1.659	9.467	2.927	2.791
SPRY1	2.079	12.391	4.074	4.901
BCL2L1	31.375	113.956	39.402	41.852
XCL2	0.056	70.747	0	2.074
KCNK1	10.828	36.893	10.967	13.139
CREB5	0.031	4.533	0.577	1.966
SLAMF1	0.042	5.663	0.217	0.275
MIR6775	12.409	50.527	19.643	21.57
ARHGEF3	6.146	31.337	8.637	8.674
CSF2	0	23.497	0	0.314
AGK	14.395	39.056	14.029	14.306
CD97	10.97	49.481	19.394	21.496
IRF4	0.034	8.439	0.423	2.092
NPDC1	3.008	8.759	4.328	5.64
CCL20	0	59.393	0	1.279
CABP1	1.702	5.834	1.201	1.689
ZNF704	0.107	4.985	0.014	0.183
CD70	1.101	61.146	3.005	7.606
TDRD9	1.428	25.902	1.245	1.888
IL21R-AS1	0.048	23.616	1.927	3.991
ATP6V1B2	33.172	129.395	50.672	60.661
CCL3L3	0	6.524	0	0.079
TM4SF1	0	3.268	0.439	0.907
RGS16	0.077	29.102	1.172	5.595
IL3	0	229.054	0.062	6.298
LAX1	8.344	24	8.052	7.578
SERPINB2	0.28	85.882	2.905	14.103
CCDC64	5.477	33.545	10.044	11.039
LRRC8B	2.89	28.699	3.555	4.298
STAT5A	1.417	9.105	3.347	3.055
SHC4	0.069	4.391	0.663	0.686
ASAP1	4.678	18.37	7.765	9.052
PTPN22	8.928	45.38	6.37	5.119
TNFRSF4	2.597	31.683	2.933	11.916
NDFIP1	19.638	54.615	22.485	24.432
LAMP3	8.064	31.61	6.412	9.828

LOC729603	1.468	5.071	2.179	3.329
PKIA	6.444	49.019	13.962	18.138
CD55	3.587	39.451	6.015	12.082
PHEX	0.188	17.377	0.213	0.786
GRK5	5.451	21.464	8.481	8.975
FMNL2	2.395	6.813	2.726	3.019
CCL4L2	0	12.758	0.087	0.328
CRIM1	0.268	3.029	0.305	0.333
ISM1	0.798	56.893	0.726	3.737
FBXO34	11.735	48.015	14.612	18.386
MAP2K3	21.676	144.057	41.815	43.237
TBC1D20	11.844	35.808	17.7	18.427
CCL1	0	9.86	0	0.415
PIM3	37.434	180.382	42.827	72.895
RDH10	4.125	19.889	5.856	5.524
CRTAM	0.104	100.175	3.325	9.51
PELI1	5.755	16.085	8.175	8.465
CCND2	0.321	6.436	0.558	0.99
CXCR3	0.604	14.322	0.503	1.803
IRF8	1.168	14.813	1.19	1.914
REL	2.654	20.219	4.749	5.699
CCL4	0	247.287	0	4.062
KCNK5	3.84	14.865	3.906	4.904
PLK3	4.432	14.807	5.257	7.036
TNS3	0.008	13.196	0.678	1.418
CD27-AS1	3.088	14.958	5.11	4.613
C3	0.006	3.907	0.489	1.329
CCL4L1	0	13.27	0.087	0.328
CCL3L1	0	6.448	0	0.079
TNFSF9	1.393	7.107	2.568	2.651
CST7	3.315	14.15	2.338	3.175
FOXO1	1.167	8.247	1.77	3.736
ATG14	5.727	30.239	7.863	11.252
NR4A2	0	10.173	0.04	0.356
C10orf54	0.84	7.506	0.382	0.487
MYO1D	4.114	11.118	4.59	5.995
CD27	2.525	27.462	3.098	3.4
